# RNA-Seq analysis of the pyloric caecum, liver, and muscle reveals molecular mechanisms regulating fillet color in rainbow trout

**DOI:** 10.1186/s12864-023-09688-5

**Published:** 2023-09-28

**Authors:** Ridwan O. Ahmed, Ali Ali, Tim Leeds, Mohamed Salem

**Affiliations:** 1 Department of Animal and Avian Sciences, University of Maryland, College Park, MD 20742 USA; 2 Department of Agriculture Kearneysville, National Center for Cool and Cold Water Aquaculture, United States, Agricultural Research Service, Kearneysville, WV 25430 USA

**Keywords:** Aquaculture, Antioxidant, Beta-carotene, Micellization, Pathways, Pigmentation

## Abstract

**Background:**

The characteristic pink-reddish color in the salmonids fillet is an important, appealing quality trait for consumers and producers. The color results from diet supplementation with carotenoids, which accounts for up to 20–30% of the feed cost. Pigment retention in the muscle is a highly variable phenotype. In this study, we aimed to understand the molecular basis for the variation in fillet color when rainbow trout (Oncorhynchus mykiss) fish families were fed an Astaxanthin-supplemented diet. We used RNA-Seq to study the transcriptome profile in the pyloric caecum, liver, and muscle from fish families with pink-reddish fillet coloration (red) versus those with lighter pale coloration (white).

**Results:**

More DEGs were identified in the muscle (5,148) and liver (3,180) than in the pyloric caecum (272). Genes involved in lipid/carotenoid metabolism and transport, ribosomal activities, mitochondrial functions, and stress homeostasis were uniquely enriched in the muscle and liver. For instance, the two beta carotene genes (BCO1 and BCO2) were significantly under-represented in the muscle of the red fillet group favoring more carotenoid retention. Enriched genes in the pyloric caecum were involved in intestinal absorption and transport of carotenoids and lipids. In addition, the analysis revealed the modulation of several genes with immune functions in the pyloric caecum, liver, and muscle.

**Conclusion:**

The results from this study deepen our understanding of carotenoid dynamics in rainbow trout and can guide us on strategies to improve Astaxanthin retention in the rainbow trout fillet.

**Supplementary Information:**

The online version contains supplementary material available at 10.1186/s12864-023-09688-5.

## Background

Carotenoids, also called tetraterpenoids, are organic molecular pigments synthesized by plants, certain bacteria, algae, and fungi [1]. Their primary function is to absorb light energy during photosynthesis and to provide photoprotection [2]. Carotenoids are structurally classified as either carotene (those without oxygen) or xanthophyll (which contains oxygen). They absorb light of wavelength between 400 and 550 nanometers, conferring yellow, orange, or red coloration [1]. They give characteristic color to plants like carrots, pumpkins, and tomatoes. In animals, carotenoids are used for pigmentation, as a precursor to vitamin A, as an antioxidant, and to enhance immune response [3, 4].

In the natural marine habitat, salmonid fish, including rainbow trout and Atlantic salmon, feed on sea algae and small crustaceans, giving the muscle pink/reddish coloration characteristics. In aquaculture, synthetic carotenoids, especially Astaxanthin, are added as feed additives to provide similar fillet coloration. This characteristic reddish/pink fillet coloration is an important quality criterion that can influence consumers purchasing decisions [5]. It has been observed that only a small proportion (2–22%) of the supplied Astaxanthin is deposited and retained in the muscle of rainbow trout [6, 7]. The large disparity between the digestibility of Astaxanthin in salmonids (approximately 30–50%) [8] and muscle retention in rainbow trout (2–22%) [6] suggests that several organs between the intestine, where carotenoids are absorbed, and muscle, where it is deposited, are crucial to the understanding of carotenoid metabolism. The diet, genetics, sexual maturity, and sex are factors that influence fillet color in salmonids [9, 10]. At a younger age, Astaxanthin is preferentially deposited in the muscle. Upon sexual maturation, carotenoids are relocated from muscle to the eggs (in females) and skin (in males) [11, 12].

Rainbow trout is the most cultivated, cold/cool freshwater fish in the United States [13], reared mainly to produce fillets. It supplies human protein with low saturated fat, cholesterol content, and high omega-3 fatty acids. Understanding the mechanism of carotenoid metabolism in rainbow trout is crucial to devising strategies to improve muscle retention of the supplied Astaxanthin. Most of our understanding of carotenoid metabolism comes from using beta-carotene in human studies and a few studies from fish. Due to their hydrophobic nature, carotenoids are closely associated with fatty acids and transported with them in the intestine and blood [14, 15]. There is, therefore, a strong link between carotenoid metabolism and fat metabolism through the uptake, transport, and delivery of both compounds. Similarly, essential features of carotenoid absorption, metabolism, and transport are similar in both salmonids and mammals [16]. Increasing dietary lipid levels improved Astaxanthin deposition and retention in rainbow trout and Atlantic salmon fillet [17, 18]. Studies have shown that the gastrointestinal tract of salmon and rainbow trout consists of several regions, with the pyloric caecum, rather than the stomach or hindgut, playing the most prominent role in the digestion, absorption, and metabolism of lipid and Astaxanthin [19–21]. Several enzymes involved in Astaxanthin’s absorption, metabolism, and transport are expressed in the pyloric caeca [22]. Several genetic factors influence carotenoid digestion in the intestine, including genes for digestive enzymes and bile acid formation that assist in carotenoid micellization, uptake of carotenoid, and transport [23, 24].

It was previously thought that the absorption of carotenoids in the intestine is passive [25]. Recent studies have shown that several proteins, including scavenger receptor class B (SCARB1), cluster of differentiation 36 (CD36), NPC1L1 (Niemann–Pick C1-like 1), and ABCA1 (ATP-Binding Cassette A1) [25, 26] facilitate carotenoid uptake in the intestine. Within the intestine, several proteins, including beta-carotene oxygenase, retinol dehydrogenases, and retinol-binding proteins, are reported to metabolize carotenoids [9, 27–29]. Genomic studies including Genome-wide association studies (GWAS) have identified genetic variants within *beta-carotene oxygenase 1* (*bco1*) and *beta-carotene oxygenase 1 like* (*bco1l)* that are associated with the variation of fillet color in salmon [28, 30]. The unmetabolized carotenoids and retinol from the intestine are transported to the liver for further metabolism. The liver is the main metabolic organ for carotenoids in salmonids [31–33]. The exact mechanism of carotenoid transport in the blood, liver uptake, and metabolism in rainbow trout remain a subject of continuous research. Studies have shown that Astaxanthin not metabolized in the liver is repackaged into very low-density lipoproteins (VLDL) and sent into the blood again for transport and deposition in the muscle [16, 34]. Astaxanthin in the muscle boosts the fillet color and confers the reddish-pink fillet coloration characteristics of salmonids. During sexual maturation, carotenoids from the muscle are transferred to the skin and gonads [10].

In this study, we used RNA-Seq analysis to elucidate on the mechanisms involved in the absorption, metabolism, and deposition of Astaxanthin in the muscle tissue of rainbow trout with a view to select for rainbow trout fish families with a better ability to retain Astaxanthin. As a result of their role in carotenoid metabolism in the literature, the pyloric caecum, liver, and muscle tissue were selected for this study. This will shed light on the molecular basis for the development of divergent intensity of fillet coloration in rainbow trout fish fed the same diet and reared under the same experimental conditions. Unlike most of the other studies in fish where one group is fed Astaxanthin and the other group is fed a diet devoid of Astaxanthin, the white and red fillet group in this study were both fed Astaxanthin in their diet to reflect the actual inability/lesser ability of some fish to utilize carotenoid for fillet color and the DEGs modulating such. Similarly, Astaxanthin is suggested to be better utilized in rainbow trout than in Atlantic salmon because of a greater digestibility of Astaxanthin (91–97% vs. 45–74%) and greater deposition of Astaxanthin in the flesh [35].

## Methods

### Ethical statement

Husbandry practice and experimental procedures at the facility were approved by the IACUC animal study protocol of the University of Maryland, College Park, protocol number 1593175-6.

### Rainbow trout population, experimental design, treatments, and sampling

This study was carried out using rainbow trout from a muscle yield genetic selection line developed at the National Center for Cool and Cold water aquaculture (NCCCWA). This line started as a growth-selected line in 2002 and underwent five generations of selection for improved growth performance as described by Leeds et al. [36]. Subsequent generations were selected for muscle yield as described in Cleveland et al.(2023) [37] and Garcia et al. [38]. Fish from the 2020-year class (YC) were included in this study and thus represent 3rd -generation families from lines selected for high (ARS-FY-H) or low (ARS-FY-L) fillet yield.

### Breeding and hatching

Briefly, the fish used for this study were from 40 families (20 full-sib families each from the ARS-FY-H and -L lines) received from the NCCCWA at 322 days post-hatch and reared at the Crane Aquaculture facility of the University of Maryland, College Park. The study used all-female immature fish to minimize the age and sex effects of fillet color. The aquaculture facility uses a recirculating aquaculture system (RAS) with all water quality parameters closely monitored. The fish were fed an Astaxanthin-supplemented diet (BioTrout 4.0 mm & 6.0 mm, ~ 40ppm Astaxanthin) from Bio-Oregon at a feeding rate for approximately six months before harvest. At the age of between 450 and 485 days post-hatch, 442 fish were harvested (average body weight = 694.36 g; SD = 173.76). The fish were taken off feed a day before harvesting. Fish were euthanized using physical stunning through a blow to the skull with a blunt wooden instrument immediately followed by exsanguination. Liver, pyloric caecum, and muscle tissue were collected on the harvest day, as described below. The fish were allowed to undergo rigor mortis on ice for 48 h and manually processed into trimmed, skinless fillets on the third day. The harvest period was done for six consecutive weeks, and sampling was done so that each week, we had a representative of one to two fish per family. Sixty fish were harvested in the first and second week, while 80 were harvested in each subsequent week and 82 in the final week.

A section of the right fillet was collected from which color measurements were obtained in the region described below. A 7.5 cm×5 cm raw fillet sample was taken from a position beginning at 1.5 cm before the dorsal fin and over the lateral line in all fillets. To prevent the influence of various thickness along the fillet on the measurements taken, all samples were prepared at a uniform thickness of 1 cm. The color was measured on a skinless fillet with the Minolta Chroma Meter CR-200 device (Minolta, Model CR-300; Minolta Camera Co., Osaka, Japan), which gives readings for redness (a*), yellowness (b*), and lightness (L*). Multiple measurements were taken from the same location and the average value was used.

The saturation index (SI) (a*^2^ +b*^2^)^0.5^ was calculated for all fish, and the average SI value for each family was used to sort the 40 families into “red fillet group” for fish families of high saturation index and “white fillet group” for fish families of low saturation index value. The saturation index describes the brightness of the color [39]. The fish from families with divergent color values (red versus white) were used for this study; five families for the pyloric caecum (3 from the red fillet group versus 2 from the white fillet group, five families for the muscle (3 from the red fillet group versus 2 from the white fillet group) and eight families for the liver (4 from the red fillet group versus 4 from the white fillet group). The difference in SI between the red and the white fillet group is shown in Table 1.

**Table 1:**
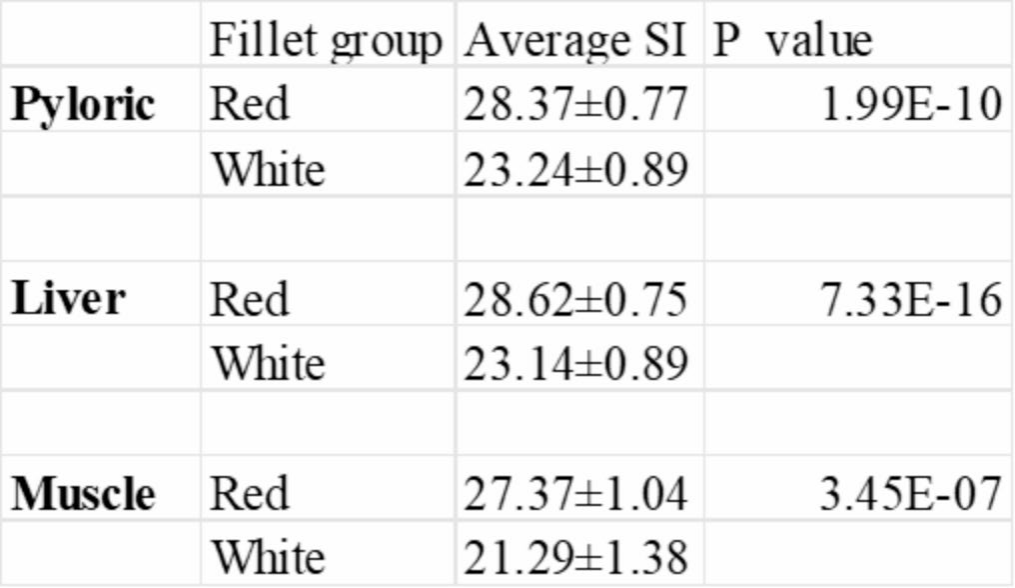
Average saturation index in the red and white fillet group in the pyloric caecum, liver and muscle

The correlation between saturation index and body weight is 0.438 in our data, but the effect of body weight did not confound our result (analysis not shown). The genetic line used for this study was selected for muscle yield, but the correlation between muscle yield and saturation index is low (0.021).

### RNA extraction

The pyloric caecum, liver, and muscle tissues were collected from selected fish samples from the red and white fillet groups. The tissue samples were immediately flash-frozen in liquid nitrogen before transferring to -80^o^c for storage. Total RNA was extracted from the tissues using the RNAzol reagent (Molecular Research Center Inc., USA) method following the manufacturer’s instructions. The concentration of RNA was measured by NanoDrop spectrophotometer Gen 5 version 2.09.2 (BioTek Instruments, Inc., USA), and the purity was estimated by the A260:A280 ratio. Gel electrophoresis was used to confirm the integrity of the extracted RNA. RNA samples selected for library preparation had an A260:A280 ratio of 1.8–2.1. RNA integrity was assessed with a ScreenTape® system (Agilent, Santa Clara, CA) and samples with RIN (RNA Integrity Number) of 7 or above were used. A total of ten (10), twenty-three (23), and sixteen (16) individual fish RNA samples were extracted from the pyloric caecum, liver, and muscle, respectively. The RNA were individually sequenced and used for this study.

### Library preparation and sequencing

Library preparation was done using the Integrated DNA Technologies (IDT) xGen RNA library preparation kit with the NEB polyA selection module (https://www.idtdna.com/pages/products/next-generation-sequencing/workflow/xgen-ngs-library-preparation/rna-library-preparation/rna-library-prep-kit#resources). The preparation is performed according to the manufacturer’s instructions. Briefly, it starts with RNA fragmentation, followed by random priming and reverse transcription to generate first-strand cDNA. This follows by tailing and adapter ligation to the 3’-end of the cDNA molecule. The last step is the PCR to increase library yield. Sequencing was performed at the Oklahoma Medical Research Foundation NGS Core, USA using an Illumina NovaSeq -S4 Instrument and paired-end 150 cycle sequencing.

### Differential gene expression analyses

The rainbow trout genome annotation was downloaded from the NCBI (GCA_002163505.1 https://www.ncbi.nlm.nih.gov/assembly/GCF_002163495.1/). Low-quality reads trimming, adapter trimming, read mapping, and differential expression analysis were performed using the CLC genomics workbench (version 22). Raw counts were used to identify differentially expressed genes (DEGs) using in-built EdgeR in the CLC genomics workbench. A gene was considered DEG when the P-adj-value < 0.05 and fold change (FC) ≥ |1.5|. Principal component analysis (PCA) was conducted to observe the clustering between samples belonging to the two phenotype groups (white fillet VS red fillet). Parameters for each step of the procedure can be accessed in supplementary file 4.

### KEGG pathways and GO analysis

Functional enrichment analysis was performed to identify KEGG (Kyoto Encyclopedia of Genes and Genomes) pathways [40] and GO (Gene Ontology) terms based on the DEGs identified against the background (expressed) genes in the pyloric caecum, liver, and muscle. The analysis was performed using ShinyGO 0.77 [41] with default setting. A cut-off of FDR-adjusted P-values of less than 0.05 was used for significant GO terms and KEGG pathways.

### Canonical pathway analysis

To investigate the molecular mechanisms underlying carotenoid absorption and utilization, the outcome from the differential expression analysis (the DEGs, fold change, and FDR-adjusted p-values) for the muscle tissue was uploaded to the Qiagen Ingenuity Pathway Analysis (IPA) software application [42]. The DEGs were categorized into related canonical pathways based on the Ingenuity pathway knowledge base (IPKB). IPA was performed to identify canonical pathways, diseases and functions, and gene networks that are most significantly enriched from our DEGs data.

## Results

### Library preparation and RNA-sequencing

A total of 1,307,592,114 raw paired-end reads (151 bp long) were sequenced for the muscle (16 samples), 1,189,353,744 raw paired-end reads, 148 bp for the liver (23 samples), and 699,709,530 raw paired-end reads, 151 bp for the pyloric caeca (10 samples). On average, 99.9% of reads per sample passed the quality control, producing 1,306,360,142 high-quality reads for muscle, 1,181,426,360 high-quality reads for the liver, and 699,314,138 high-quality reads for the pyloric caeca. The reads were mapped against the rainbow trout genome (https://www.ncbi.nlm.nih.gov/assembly/GCF_002163495.1/), producing an average of 90% mapping rate.

### Between-group clusters

Principal component analysis (PCA) showed a distinct clustering pattern between samples from the white and the red fillet groups in the liver and muscle. In addition, there is a clear separation between samples from the pyloric caecum, liver, and muscle.

### Differentially expressed genes in the pyloric

The pyloric caecum had only 272 significant (FDR P < 0.05) DEGs between the white and the red fillet groups. One hundred and twenty-six (126) of those genes were upregulated (Fold change ≥ 2.0), while 166 were downregulated (fold change ≤ -1.5). The complete list of DEGs and their fold change can be found in Supplementary Table 1.

The highest increase in mRNA expression in the DEGs between the white fillet versus the red fillet group was found in the gene encoding stonustoxin subunit beta-like (1256.28 FC), which confers immune-related functions. At the opposite end of the DEGs, the most downregulated genes are the protein *RD3* (retinal degeneration 3 (-439.07 FC) and *uromodulin* (-186.4 FC).

Genes encoding proteins involved in carotenoid/lipid absorption and transport are enriched in the red fillet group: *CD36 antigen* (-2.30 FC), *phospholipid-transporting ATPase ABCA1* (-5.75 FC, FDR-adj = 0.06). Several DEGs are involved in lipid metabolism, such as *apolipoprotein B-100* (-9.61 FC), *ELOVL fatty acid elongase 6* (-37.51 FC), *long-chain-fatty-acid–CoA ligase 3* (13.53 FC), *phospholipase A and acyltransferase 4-like* (4.79 FC), and *phospholipid phosphatase-related protein type 4* (3.29 FC). Modulated genes involved in carotenoid metabolism are *retinoic acid receptor beta* (4.37 FC), *retinol dehydrogenase 1* (2.43 FC), and *retinoic acid receptor beta* (-3.92 FC).

Among the modulated genes in the pyloric caecum encoding proteins that have functions related to immunity include *ferritin, middle subunit* in the pyloric caecum (-8.89 FC), *GTPase IMAP family member 8-like* (34.94 FC), *interferon-induced very large GTPase* 1 (16.79, 6.94 FC), *GTPase IMAP family member 7* (-6.4, -6.68, -43.58 FC), *B-cell receptor CD22-like* (-22.32 FC). Others are listed in Supplementary Table 1. There is no enriched KEGG pathway and GO terms for DEGs in the pyloric caecum.

### Differentially expressed genes in the liver

There were 3,180 significant (FDR P < 0.05) DEGs in the liver between the white and the red fillet groups. One thousand four hundred and two (1402) of those genes are upregulated, while 1778 are downregulated. The complete list of DEGs and their fold change can be found in Supplementary Table 1. The most downregulated genes encode putative per-hexamer repeat protein 5 (-4489.25 FC), pygopus homolog 1 (-82.69 FC), and follistatin A (-75.07 FC). At the opposite end, the most upregulated genes encode pentraxin-related protein PTX3 (162.32 FC), ferritin H-3 (147.72 FC), and protein FAM163B-like (93.16 FC).

Other DEGs identified in the liver involved in chylomicron uptake include those that encode for low-density lipoprotein receptor-related protein 1 (LRP1) (-1.59 FC), LRP10 (-2.18 FC), LRP6 (-1.51, -178 FC) and basement membrane-specific heparan sulfate proteoglycan core protein (-2.06, -3.56 FC). Other significant DEGs with prominent functions in carotenoid metabolism are Apolipoprotein B-100(-6.9, -1.74, -2.38 FC), Retinol binding protein 7b cellular (3.27 FC), retinol-binding protein 2 (2.84 FC), *BCO1* (1.46 FC), *BCO2* (-2.71 FC).

DEGs in the liver that encode for proteins with immune function include ferritin H-3 (147.7 FC), Heme oxygenase 2-like (-1.67 FC), interferon-induced very large GTPase 1 (-1.76, -2.08, 2.27 FC), GTPase IMAP family member 9 (5.66 FC). Others are listed in Supplementary Table 1. DEGs that encode proteins involved in stress homeostasis identified in the liver are listed in Supplementary Table 1.

### KEGG and GO terms in the liver

There are no significant KEGG and GO terms in the liver for the down-regulated genes. For upregulated genes between the white versus red fillet group in the liver, the KEGG pathways enriched are ribosome (59 genes), oxidative phosphorylation (19 genes), and protein export (5 genes).

Several GO terms are significantly enriched for this group. The most significant terms involved in the biological process, as shown in Fig. 2 and Supplementary Tables 2, include peptide biosynthetic process, translation, amide biosynthetic process, peptide metabolic process, electron transport chain, oxidative phosphorylation, ATP metabolic process, cellular respiration.

**Fig. 1:**
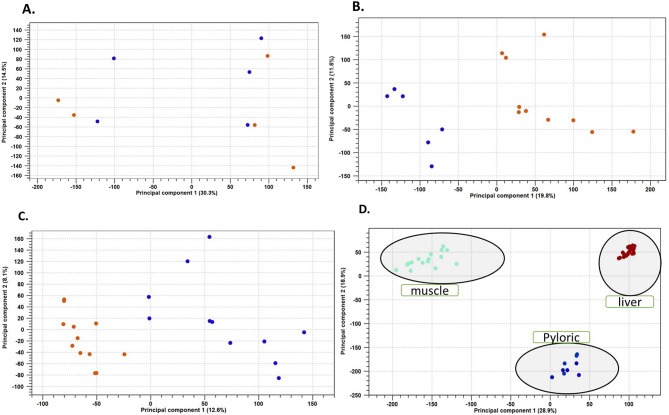
**a**: Principal component analysis (PCA) performed on the gene expression data from pyloric caeca samples showing no apparent clustering. **b**: PCA performed on the expression data of muscle samples showing the separation of the white (Low) and red (High) fillet groups. **c**: PCA performed on the gene expression data of liver samples showing the separation of the white (Low) and red (High) fillet groups. **d**: PCA performed on the expression data of pyloric caecum, liver, and muscle samples of rainbow trout. The PCA shows a clear separation of the three tissues

**Fig. 2:**
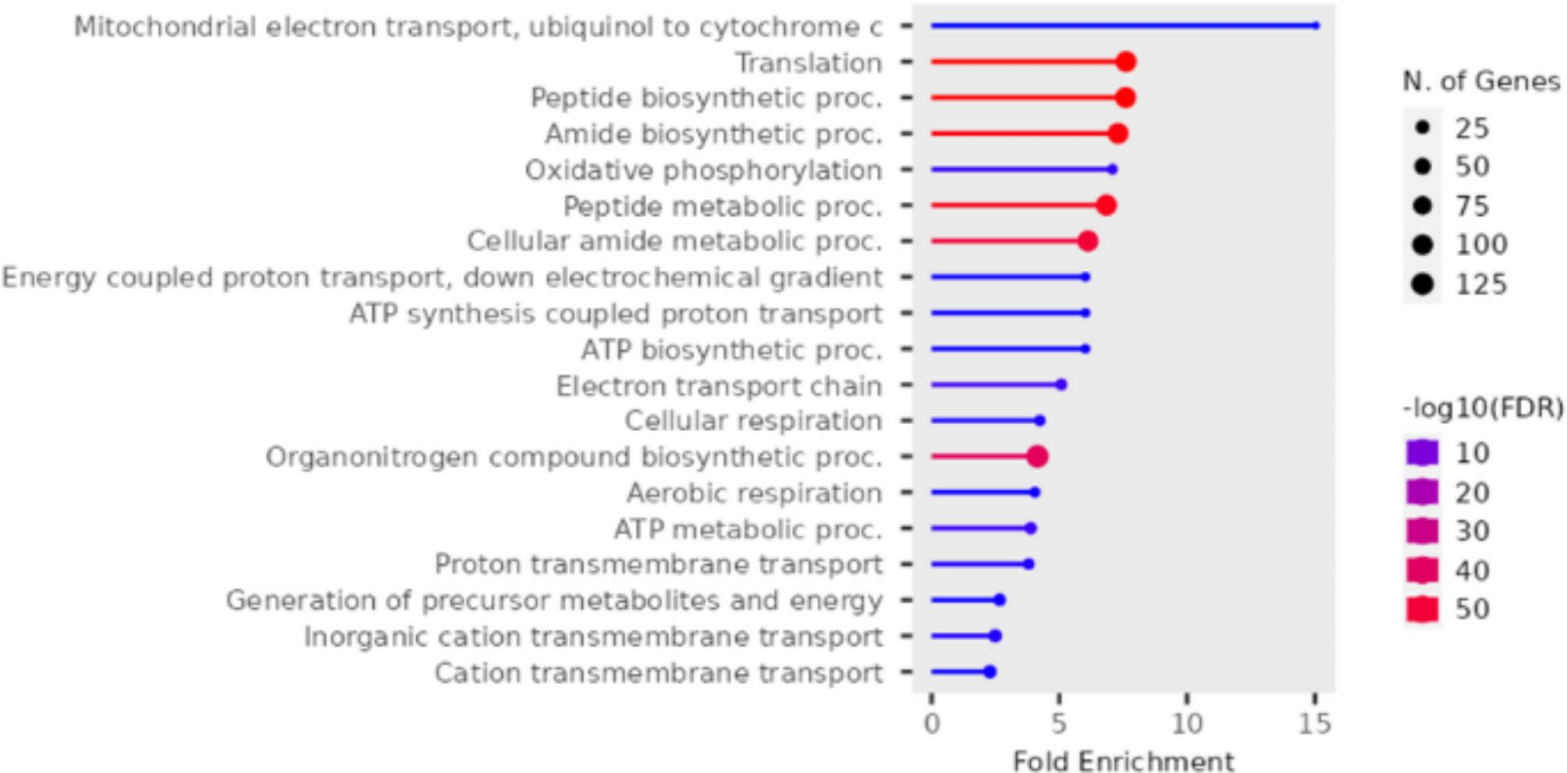
Enriched GO biological function terms for the upregulated genes in the liver comparing the white versus red fillet groups

Some of this group’s most significant cellular function terms are ribosome, ribosomal subunit, ribonucleoprotein complex, large ribosomal subunit, mitochondrial inner membrane, mitochondrion, mitochondrial envelope, and others, as presented in Supplementary Table 2.

The most significant molecular components GO terms for this group include structural constituent of ribosome, structural molecule activity, electron transfer activity, proton transmembrane transporter activity, oxidoreduction-driven active transmembrane transporter activity, RNA binding, and cyclin-dependent protein serine/threonine kinase regulator activity as presented in Supplementary Table 2.

### Differentially expressed genes in the muscle

There were 5148 significant (FDR P < 0.05) DEGs between the white and the red fillet groups. Two thousand six hundred and forty-three (2643) of those genes are upregulated (Fold change > 1.5), while 2505 are downregulated (fold change > -1.5). The complete list of DEGs and their fold change can be found in Supplementary Table 1. The most upregulated genes between the white versus red fillet group in the muscle encode digestive enzymes with immune functions: acidic mammalian chitinase (728.07, 575.56 FC) and gastric chitinase (565.2 FC).

The downregulated genes with the most significant fold change encode proteins with stress homeostasis function. They include heat shock protein 30 (-297.82, -151.27, -136.33, -135.52 FC), nucleotide triphosphate diphosphatase NUDT15 (-268.43 FC), and asporin (LRR class 1) (-942.47 FC).

DEGs involved in carotenoid metabolism include *albumin 1* (98.85, 86.42 FC, *low-density lipoprotein receptor-related protein 2b* (*LRP2B*) (-3.27 FC), *low-density lipoprotein receptor-related protein 2a* (*LRP2A*) (-3.37 FC), *low-density lipoprotein receptor-related protein 1Ba* (*LRP1BA*) (-11.73 FC), *low-density lipoprotein receptor-related protein 4* (*LRP4*) (2.61 FC), *low-density lipoprotein receptor adaptor protein 1a* (*ldlrap1a*) (2.18 FC), *BCO2* (10.46), and *BCO1* (3.04). Coronins (*coronin-2B* (-1.61 FC) and *Coronin-1 C* (-18.77 FC)) are involved in the transport of Astaxanthin within the muscle cells.

Several DEGs in the muscle encode proteins involved in actomyosin structure organization. They are listed in Supplementary Table 1.

DEGs that encode for proteins involved in stress homeostasis in the muscle are tumor necrosis factor receptor superfamily member 14 (4.26 FC), glutathione peroxidase 1 (gpx1) (-1.75, -2.71 FC), probable glutathione peroxidase 8 (-2.29 FC), superoxide dismutase (-1.9 FC), Hsp30 (-5.62, -11.59, -53.05, -55.93, -92.8, -135.53, -136.63, -151.27, -92.8, -297.82 FC) and hsp70(-3.02 FC). Others are listed in Supplementary Table 1.

DEGs involved in immune response are *ferritin, middle subunit* (-1.96, -2.01, -2.4, -2.85, -3.28,-3.5, -6.53 FC), *Cathepsin Bb* (-1.68 FC), *cathepsin K* (-1.78, -5.88, 5.75 FC), *GTPase IMAP family member 7* (3.78, 11.1, -5.06 FC), *Ladderlectin* (-8.15 FC), *tripartite motif-containing protein 35-like (5.77 FC).* Others are listed in Supplementary Table 1.

### Canonical pathways

To investigate the molecular mechanisms underlying carotenoid absorption and utilization in the muscle, the DEG list in the muscle was submitted to Ingenuity Pathway Analysis (IPA) core analysis from Qiagen software. The DEGs were categorized into related canonical pathways based on the Ingenuity pathway knowledge base (IPKB). IPA was performed to identify canonical pathways and ‘diseases and functions’ that are most significantly enriched from our DEGs data.

**Fig. 3:**
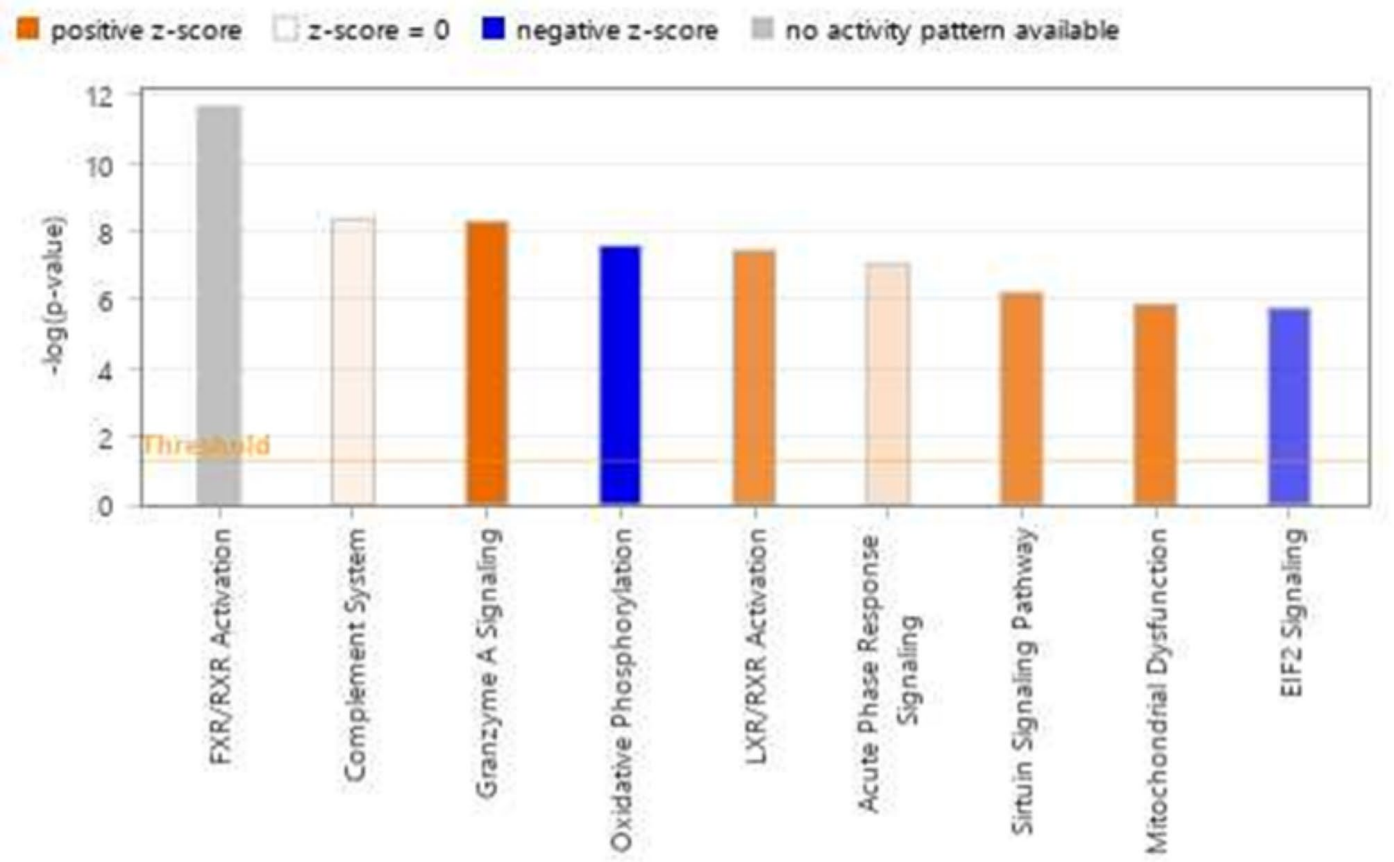
The topmost significant canonical pathways for the muscle DEGs

The top enriched canonical pathways in the muscle with a p-value less than 0.05 are presented in Fig. 3. The FXR/RXR and LXR/RXR activation pathways are the most significantly enriched pathways. Others are oxidative phosphorylation and mitochondria dysfunction.

### Disease and function analysis

**Fig. 4:**
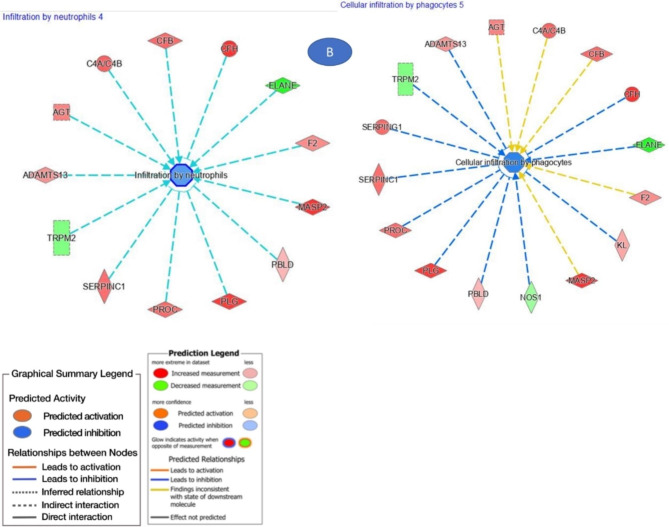
Modulated diseases and functions from the DEGs in the muscle (a) Genes related to lipid metabolism (b) Genes related to immunity

The IPA also categorizes DEGs into related diseases and functions. Some diseases and functions with a p-value less than 10^− 5^ and the number of representative genes involved are listed in Fig. 4; Table 2. The full list of all diseases and functions categories and the genes involved is included in Supplementary Table 3. Those functions related to lipid metabolism include disorder of lipid metabolism (P-value 1.40E-08) which is activated in the white fillet group, and uptake of lipids (P-value 1.59E-05) which is inhibited in the white fillet group. Others related to immunity with a p-value less than 0.05 include infiltration by neutrophils and cellular infiltration by phagocytes, both inhibited in the white fillet group.

**Table 2:**
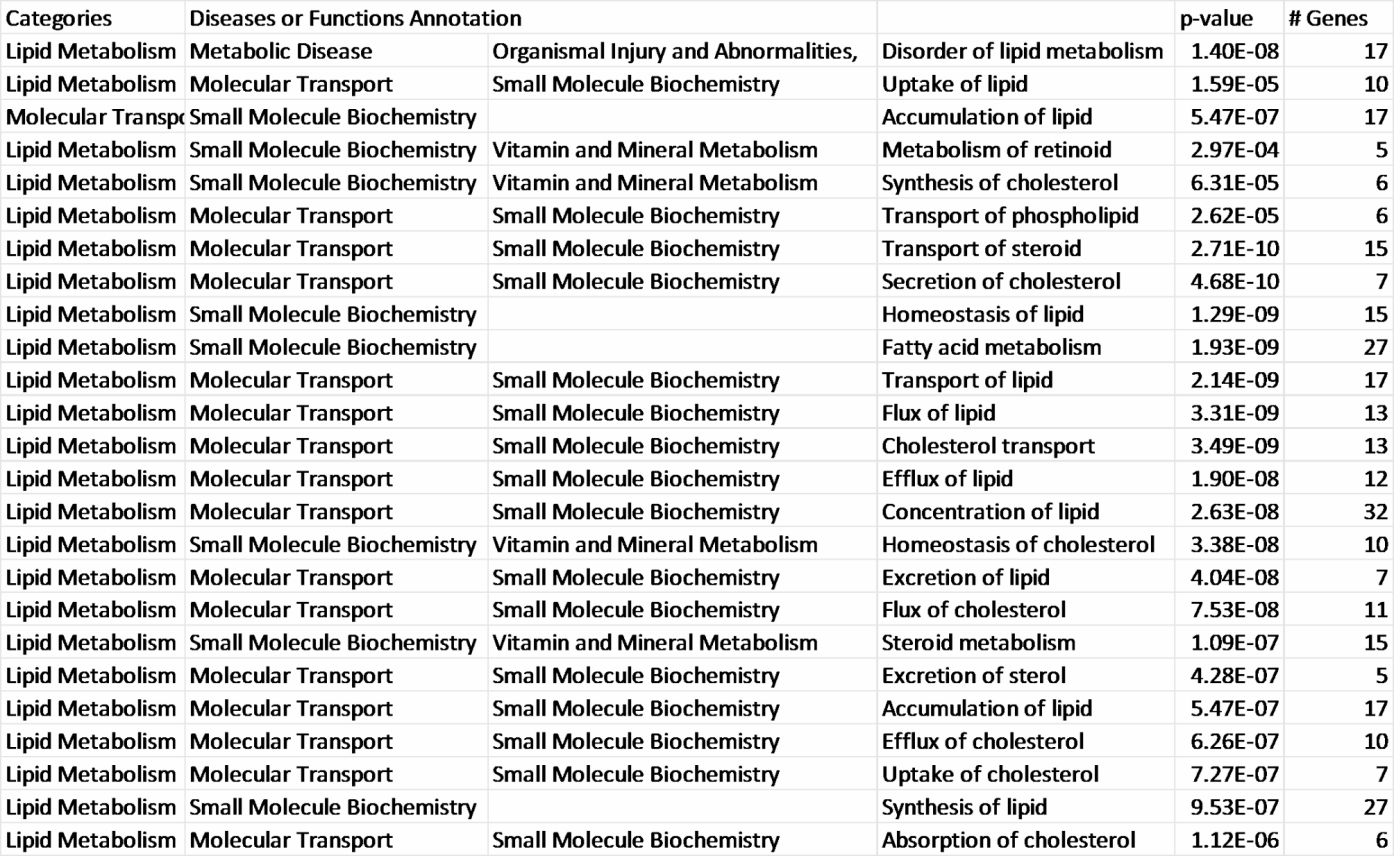
Top diseases and functions annotations identified by IPA

### KEGG pathways and GO terms in the muscle

**Fig. 5:**
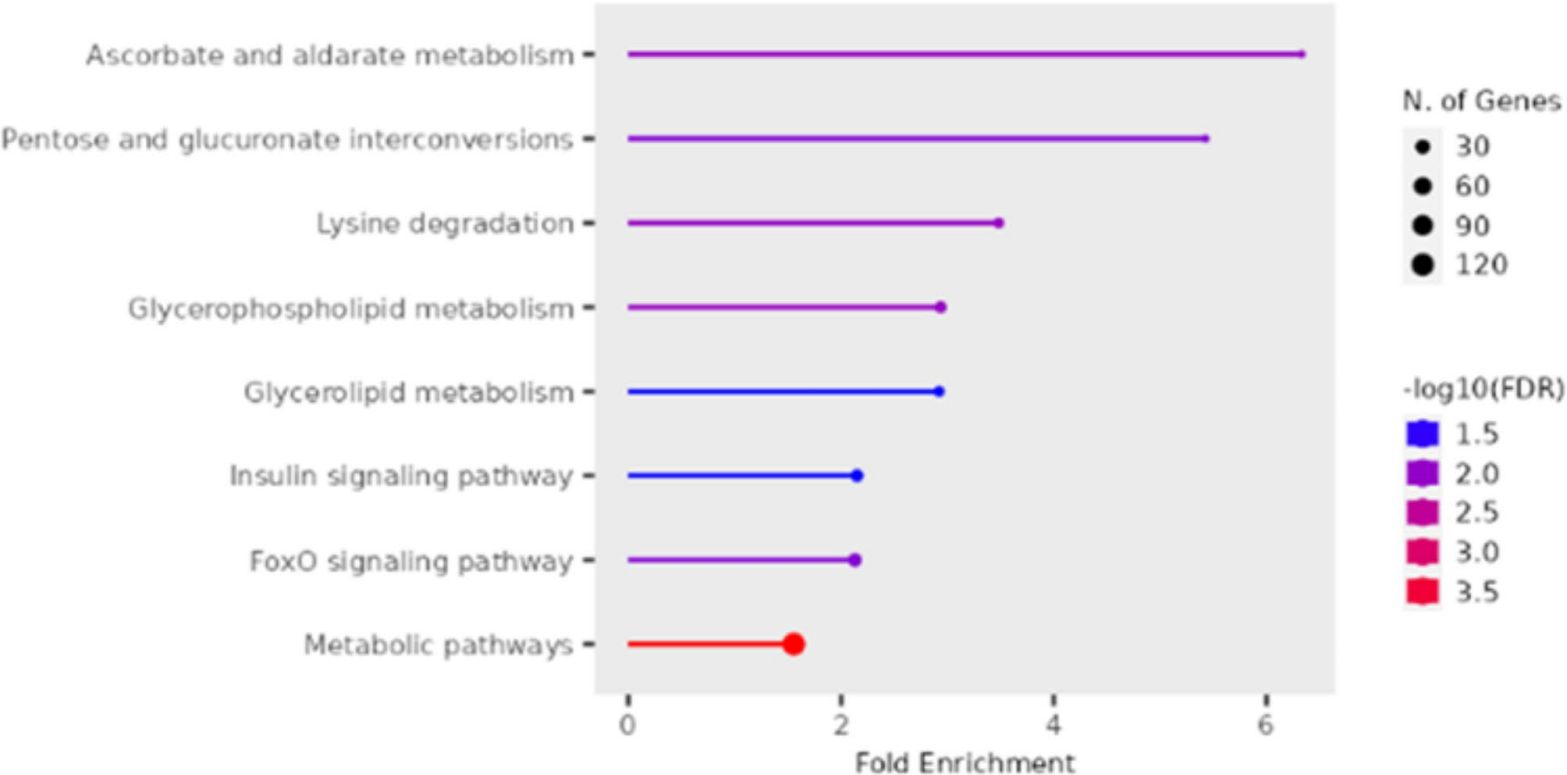
Enriched KEGG pathways for upregulated genes in the muscle comparing the white versus red fillet groups [40]

In the muscle, the KEGG pathways enriched for the upregulated genes in the white versus red fillet group, as shown in Fig. 5, include metabolic pathways (122 genes), ascorbate and aldarate metabolism (6 genes), lysine degradation (11 genes), glycerophospholipid metabolism (15 genes), pentose and glucuronate interconversions (6 genes), FoxO signaling pathway (23 genes), glycerolipid metabolism (10 genes) and insulin signaling pathway (18 genes).

**Fig. 6:**
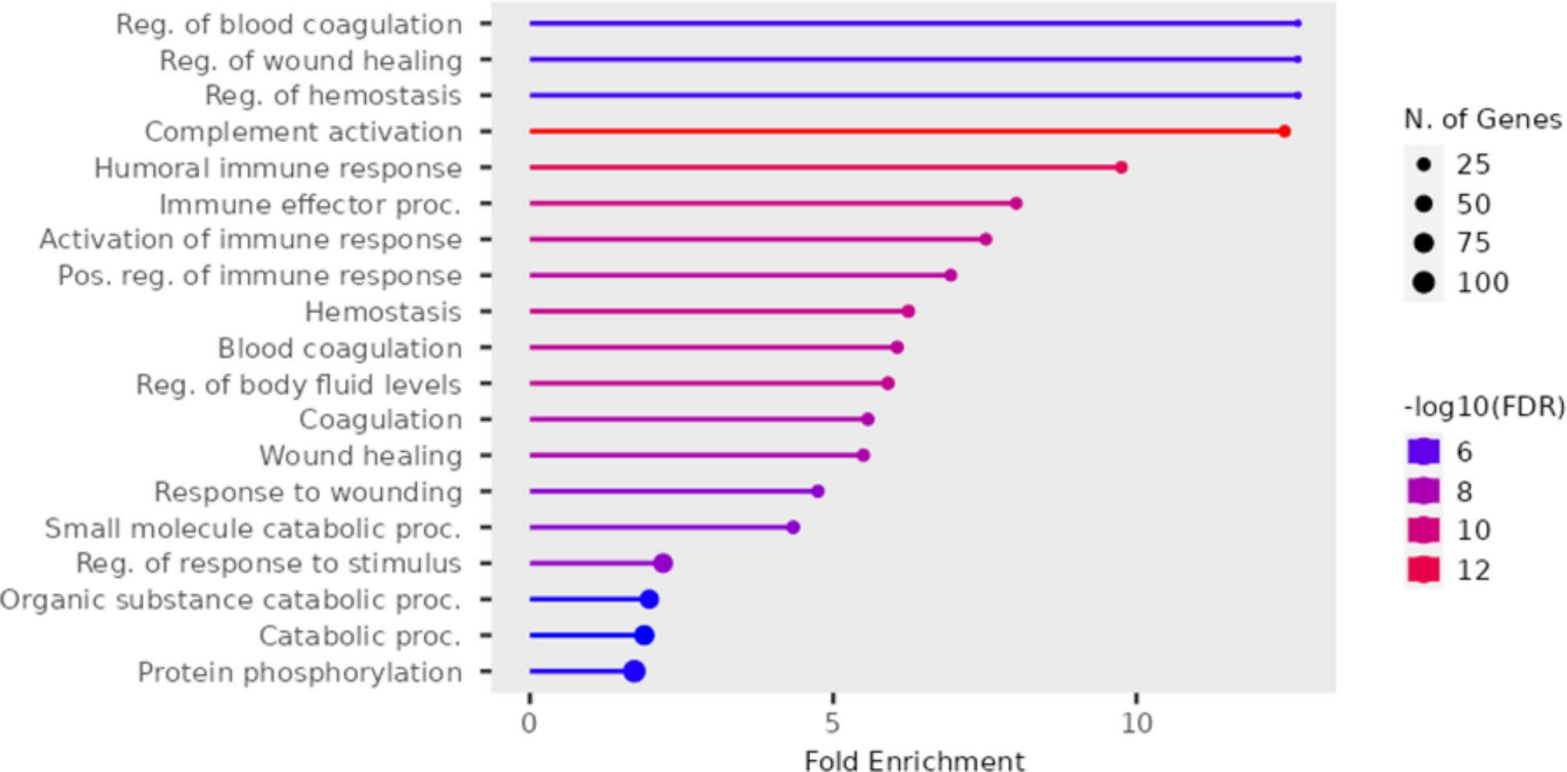
Enriched GO Biological process terms for the upregulated genes in the muscle comparing the white versus red fillet groups

Several GO terms are significantly enriched for this group. The most significant terms involved in the biological process, as shown in Fig. 6 and Supplementary Tables 2, include complement activation, humoral immune response, immune effector process, hemostasis, activation of the immune response, regulation of body fluid levels, blood coagulation, positive regulation of immune response, coagulation, wound healing, positive regulation of immune system process, regulation of response to stimulus, response to wounding, regulation of blood coagulation, regulation of hemostasis, phosphorylation, lipid transport, lipid localization, regulation of immune response, and regulation of stress response.

The most significant GO terms for cellular components in this group, as shown in Supplementary Tables 2, include extracellular space, MLL1/2 complex, MLL1 complex, histone methyltransferase complex, SWI/SNF superfamily-type complex, ATPase complex, methyltransferase complex, nucleoplasm, and transferase complex.

Some of the most significant molecular function terms for this group are endopeptidase inhibitor activity, peptidase regulator activity, endopeptidase regulator activity, peptidase inhibitor activity, enzyme regulator activity, serine-type endopeptidase inhibitor activity, transition metal ion binding, protein serine/threonine kinase activity, protein kinase activity, oxidoreductase activity, acting on the CH-NH group of donors, NAD or NADP as acceptor, histone-lysine N-methyltransferase activity, histone methyltransferase activity, transferase activity, and transferring phosphorus-containing groups as listed in Supplementary Table 2.

**Fig. 7:**
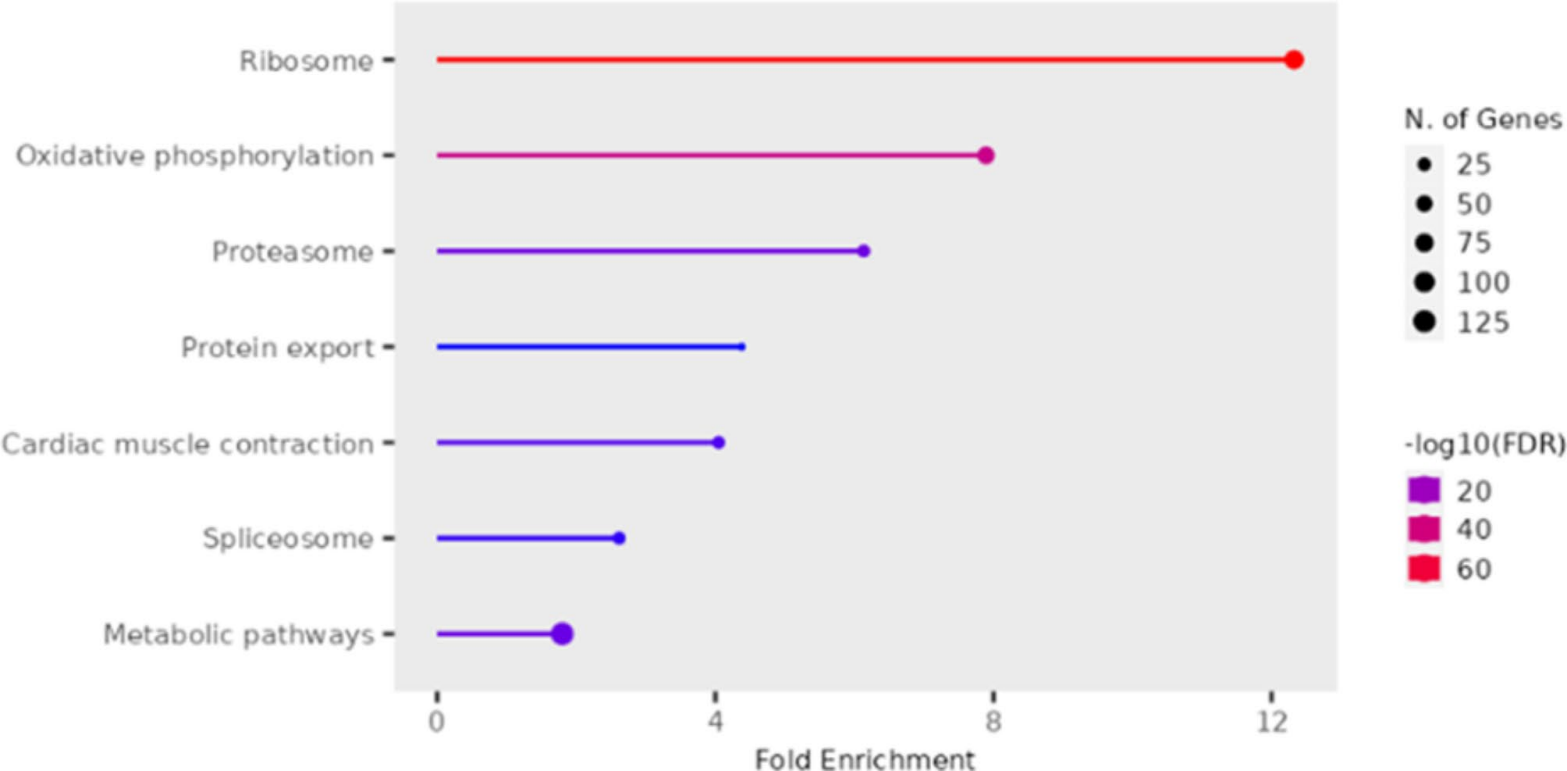
Enriched KEGG pathways for the downregulated genes in the muscle comparing the white versus red fillet group [40]

For the downregulated genes between the white versus red fillet group, the KEGG pathways enriched are ribosome (85 genes), oxidative phosphorylation (61 genes), proteasome (21 genes), cardiac muscle contraction (20 genes), metabolic pathways (131 genes) and spliceosome (21 genes) as shown in Fig. 7.

**Fig. 8:**
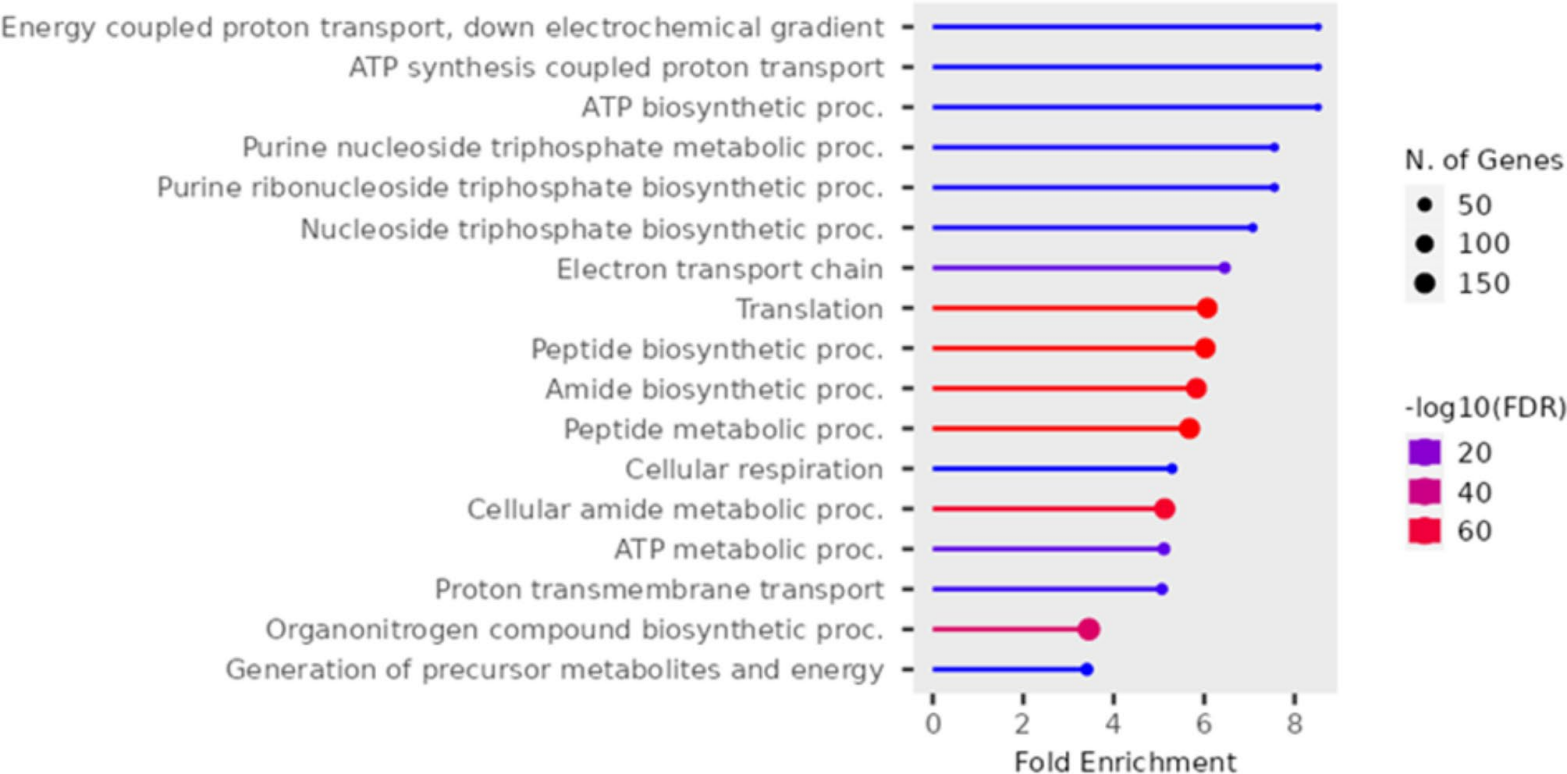
Enriched GO biological process for the downregulated genes in the muscle comparing white versus red fillet group

Several GO terms are significantly enriched for this group. The most significant terms involved in the biological process, as shown in Fig. 8 and Supplementary Tables 2, include peptide biosynthetic process, translation, peptide metabolic process, amide biosynthetic process, electron transport chain, ATP metabolic process, proton transmembrane transport, nucleoside triphosphate biosynthetic process, purine nucleoside triphosphate metabolic process, ATP biosynthetic process, oxidative phosphorylation, respiratory electron transport chain, mitochondrial respiratory chain complex assembly, and mitochondrial ATP synthesis coupled electron transport.

Most significant cellular components include ribosome, non-membrane-bounded organelle, mitochondrion, mitochondrial inner membrane, ribosomal subunit, ribonucleoprotein complex, mitochondrial envelope and respirasome (Supplementary Table 2).

The most significant molecular function GO terms are structural constituent of ribosome, oxidoreduction-driven active transmembrane transporter activity, proton transmembrane transporter activity, electron transfer activity, cytochrome-c oxidase activity, oxidoreductase activity, acting on a heme group of donors, primary active transmembrane transporter activity, threonine-type endopeptidase activity, RNA binding, NADH dehydrogenase activity, NAD(P)H dehydrogenase (quinone) activity, NADH dehydrogenase (ubiquinone) activity, calcium ion binding, inorganic molecular entity transmembrane transporter activity, ion transmembrane transporter activity, phospholipase inhibitor activity, lipase inhibitor activity, active transmembrane transporter activity and transporter activity (Supplementary Table 2).

## Discussion

This study investigated molecular mechanisms influencing fillet color and the utilization of carotenoids in rainbow trout. We compared the transcriptome profile in the pyloric caecum, liver, and muscle of rainbow trout groups with ‘white’ versus ‘red’ fillets. Fish in both groups were fed Astaxanthin supplemented diet. Carotenoid absorption mainly occurs in the pyloric caecum, followed by liver metabolism and muscle deposition. More DEGs exist in the muscle and liver than in the pyloric caecum. Metabolism of carotenoids is closely linked to lipid and fatty acid metabolism [43], and in agreement with this, several DEGs are involved in lipid metabolism and transport. DEGs involved in carotenoid/lipid absorption and transport were identified in the pyloric caecum. DEGs involved in mitochondrial function, ribosomal activities, and protein functions are identified in the liver and muscle. In the muscle, lipid metabolism disorders and lipid uptake inhibition were observed in the white fillet group, which might inhibit the retention of Astaxanthin. Other DEGs have immune-related functions and are involved in stress homeostasis (Supplementary Table 1).

### Pyloric caecum

The stonustoxin subunit beta-like (1256.28 FC) gene is the most upregulated gene in the pyloric caecum of the white fillet group in this study. LeBlanc et al. [44] identified stonustoxin as one of the innate immune response genes upregulated in the head-kidney of Atlantic salmon injected with salmon anemia virus (ISAV) relative to the control Atlantic salmon. At the opposite end of the DEGs, the most downregulated genes are the protein *RD3* (retinal degeneration 3 (-439.07 FC) and *uromodulin* (-186.4 FC). Uromodulin protects against urinary tract infections and is an antioxidant [45]. It is also known that carotenoid pigments are found in the retina, acting as an antioxidant and free radical scavenger to reduce oxidative stress-induced damage [46].

### Absorption of carotenoids

Carotenoids are insoluble in water and are thus absorbed and transported through their incorporation into lipids and fatty acids [43] by forming mixed micelle in the gut after their release from the ingesta [47]. Differences in the pigmentation between the white and red fillet groups could result from the difference in their ability to absorb, metabolize and utilize carotenoids inside the pyloric caecum. A higher content of lipase and bile fosters micellization [48], as well as the presence of lipids in the diet [49]. In this study, genes such as lipid droplet assembly factor, apolipoprotein B-100 (-9.6 FC), and ELOVL fatty acid elongase 6 (-37.51 FC) are downregulated in the pyloric caecum of the white fillet group, probably conferring advantage to the red fillet group in their ability to form mixed micelle with Astaxanthin. Genetic polymorphisms in the ELOVL fatty acid elongase 2 plays a significant role in carotenoid absorption in human [50–52]. Although long-chain-fatty-acid–CoA ligase 3 (13.54 FC) and fatty-acid amide hydrolase 1 (4.75 FC) are also enriched in the white fillet group. The *FXR* (farnesoid X receptor) and *HNF4-alpha* function in tandem to regulate bile acid synthesis [53]. Bile acids facilitate intestinal absorption and transport of lipids and other nutrients. The hepatocyte nuclear factor 1-alpha (*HNF4-alpha*) (2.29 FC) is upregulated in the pyloric caecum of the red fillet group. This upregulation might be advantageous to micellization and absorption of Astaxanthin and lipids in the red fillet group. The *PPARα* (peroxisome proliferator-activated receptor α) is also reported to regulate bile acid synthesis [53].

The mixed micelles then diffuse through the mucus layer of the enterocyte, ready for absorption. Carotenoid absorption in the intestine was previously considered as a passive transport [54]. However, identifying several membrane proteins/transporters, such as CD36 and scavenger receptors, suggests otherwise [55, 56]. We identified that the *cd36 antigen* (-2.3 FC) is expressed less in the white fillet fish’s pyloric caecum than those in the red fillet group. The *cd36 antigen* plays a role in carotenoid uptake by the intestinal cells [57]. They facilitate the absorption of carotenoids across the brush border membrane of the enterocyte. This suggests that the red fillet group possesses a more remarkable ability to absorb and transport Astaxanthin. *CD36* was upregulated in the pyloric caecum of Atlantic salmon fed supplemented Astaxanthin compared to fish fed a control diet [22].

The white fillet group showed higher expression in *NPC1L1 Intracellular cholesterol transporter 1*, as observed in the white Chinook fish in Madaro et al. [27]. The NPC1L protein specializes in cholesterol absorption into the cell and plays a critical role in lipid metabolism [58]. It has been identified as one of the proteins involved in the absorption and transport of carotenoids in human and fish studies [27, 47]. It might be that absorption of carotenoids with *NPC1L* is not as efficient as with *CD36* protein that is upregulated in the red fillet group.

### Metabolism of Astaxanthin inside the enterocyte

After the uptake of the carotenoid across the apical brush border membrane and inside the enterocyte, it is reported to undergo intracellular metabolism before traveling to the basolateral side of the cell [47]. Beta-carotene 15,15’- oxygenase 1 (*BCO1*) and beta, beta-carotene 9,10’- oxygenase (*BCO2*) cleave beta-carotene inside the enterocyte. *BCO1* cleaves beta-carotene at its central double bond to yield retinal (a precursor of retinol and retinoic acid), while beta-carotene undergoes eccentric cleavage by *BCO2* to yield apocarotenoids [59]. Neither enzyme are enriched in the pyloric caecum in this study.

Retinol dehydrogenases are a group of enzymes involved in metabolizing vitamin A and carotenoids [60, 61]. After cleavage of carotenoids by *BCO1* and *BCO2*, they are converted into all-trans-retinoic acid and retinol. All trans-retinoic acid and retinol are further converted into retinal by retinol dehydrogenases [60]. The short-chain dehydrogenase gene family can also achieve this oxidation to retinal in humans [62–64]. Retinol dehydrogenase 1 (*rdh1*) (2.43 FC) and dehydrogenase/reductase (SDR family) member 12 (3.60 FC) are upregulated in the pyloric caecum of the white fillet group in this study. This might suggest the availability of more retinoic acid and its derivatives in the white fillet group due to the activities of beta-carotene 15,15’- oxygenase 1 (*BCO1*) and beta, beta-carotene 9,10’- oxygenase (*BCO2*). Similarly, retinoic acid receptor beta (4.37FC) is upregulated in the white fillet group. Contrary to our findings, *rdh3, rdh8*, and retinal dehydrogenase 2 (*aldh1a2*) were upregulated in the pyloric caecum of the Atlantic salmon fed Astaxanthin-supplemented diet compared to those on the non-Astaxanthin diet [22]. This discrepancy might be due to differences in the design of the two experiments, i.e., absence of supplemental Astaxanthin in one group of fish in Schmeisser et al. [22] compared to our study, where both the white and red fillet groups were fed supplemental Astaxanthin. Retinol dehydrogenase-7 was identified in a genome-wide association study as one of the genes influencing fillet color in rainbow trout [5].

Carotenoids inside the enterocyte that *BCO1* and *BCO2* do not metabolize are transported into lipoproteins and translocated to the basolateral membrane of the enterocyte [57]. This process is suggested to be carried out by fatty acid transport proteins and fatty acid binding proteins [57]. The fatty acid-binding proteins bind carotenoids and transport them within the enterocyte. Gastrotropin (Fatty acid binding protein 6) is upregulated in the pyloric caecum (8.69 FC) of the red fillet group in this study, possibly conferring an advantage to those fish in their ability to transport Astaxanthin within the pyloric caecum and muscle cells. Fatty acid transport binding proteins were upregulated in the pylorus of red Chinook fish compared to the white Chinook Salmon [27].

The uncleaved beta-carotene and retinyl esters are then incorporated into chylomicrons and transported into the liver [65]. Beta-carotene incorporation into chylomicron has been suggested to be under the regulation of enzymes microsomal TAG transfer protein (*MTP*), apoA-IV, secretion associated Ras-related GTPase 1B (*SAR1B*) and ATP binding cassette subfamily A member 1 (*ABCA1*) [47, 50]. Indeed, *ABCA1*(-5.71 FC) was downregulated in this study’s pyloric caecum of the white fillet group. *ABCA1* catalyzes the efflux of intracellular cholesterol to apolipoprotein forming high-density lipoprotein and their translocation from the cytoplasm to the extracellular membrane and into the portal blood [66]. This suggests a better ability of the red fillet group to incorporate Astaxanthin into chylomicron for transport into the liver. *Apolipoprotein B-100* (9.61 FC) is also upregulated in the pyloric caecum of the red fillet group. Apolipoprotein B-100 and apolipoprotein B-48 are components of lipoproteins that transport fat and cholesterol in the form of chylomicron and very low-density lipoproteins into the blood to the liver [67]. Madaro et al. [27] also identified upregulation of ApoA-IV in the red Chinook salmon compared to white Chinook salmon, suggesting more stabilization and transport of Astaxanthin. Single nucleotide polymorphisms close to the *Apo* genes are also associated with flesh pigmentation in Chinook salmon [68].

### Liver

On getting to the liver, chylomicrons containing carotenoids and retinyl esters are taken up by the hepatocytes through the action of cell surface several receptor proteins, including the low-density lipoprotein receptor (*LDLR*), low-density lipoprotein receptor-related protein 1 (*LRP1*), heparan sulfate proteoglycans (*HSPGs*) in human [69]. The *LRP1* (-1.59 FC), *LRP10* (-2.18 FC), *LRP6* (-1.51, -178 FC), and *basement membrane-specific heparan sulfate proteoglycan core protein* (-2.06, -3.56 FC) were downregulated in the white fillet group in this study. *LRP1b* (3.07 FC) is upregulated in the white fillet group. This synchronized expression might suggest a comparative advantage to the red fillet group in their ability to take up chylomicrons inside the hepatocyte.

Following chylomicron uptake in the liver by cell surface receptors, carotenoids and retinyl palmitate are assumed to be released into the hepatocytes [47]. Ong [70] suggested that retinyl palmitate is hydrolyzed by retinyl ester hydrolase to retinol and bound to retinol-binding protein, type 1 (*RBP1*). Retinol-binding protein 7b, cellular (3.27 FC), and retinol-binding protein 2 (2.84 FC) are upregulated in the white fillet group, suggesting higher availability of retinol from carotenoid cleavage. Retinol-binding proteins are involved in the intracellular transport of retinol. The liver’s beta carotene enzymes (BCO1 and BCO2) can cleave carotenoids into retinal [71]. While *BCO1* (1.46 FC) is upregulated in the white fillet group, *BCO2* (-2.71 FC) is downregulated, unlike in the muscle, where both are differentially expressed in the same direction. Similar findings were reported by Madaro et al. [27], where they found *BCO2* was upregulated in Chinook salmon with white phenotype, while *BCO1* was downregulated in the same phenotype group. The uncleaved fraction of carotenoids is either stored in the hepatic stellate cells or incorporated into very low-density lipoprotein (VLDL) and secreted into the blood for transport to other tissues [71, 72].

There are no significant KEGG and GO terms in the liver for the downregulated genes. The significant KEGG pathways for the DEGs in the white versus red fillet group are ribosome and oxidative phosphorylation. It is known that oxidative phosphorylation occurs in the mitochondria, and mitochondrial activities can influence fillet color. Similarly, ribosomal activities have also been associated with fillet color in fish and skin pigmentation in mice [73–75]. The most significant GO terms for this group also relate to ribosome and mitochondrial activities.

### Muscle

It has been suggested that Astaxanthin is brought to the muscle by circulating albumin [16]. A radioactive study in Atlantic salmon suggested that Astaxanthin is transported in the plasma via its association with serum albumin [76]. Vo et al. [77] also found upregulation of *albumin 2* in the gut of the more intense red-fleshed Atlantic salmon compared to the light-fleshed salmon. They suggested that this upregulation might confer an advantage to the red-fleshed salmon in their ability to absorb and transport Astaxanthin from the gut into the blood and deposit it in the liver and muscle. In the same vein, *albumin 1* (1.89 FC) is upregulated in the liver of the red fillet group, and this might aid in transporting carotenoids from the liver to the plasma and the muscle.

On getting to the muscle, Thomas & Harrison [78] proposed that the extra-hepatic tissues take up the VLDL-carotenoids in an LDL-receptor-dependent manner, requiring the tissue to express LDL-receptors. In line with that proposition, low-density lipoprotein receptor-related protein 2b (*LRP2B*) (-3.27), low-density lipoprotein receptor-related protein 2a (*LRP2A*) (-3.37 FC), low-density lipoprotein receptor-related protein 1Ba (*LRP1BA*) (-11.73 FC) are downregulated in the muscle of the white fillet group in this study. Although, low-density lipoprotein receptor-related protein 4 (*LRP4*) (2.61 FC) and low-density lipoprotein receptor adaptor protein 1a (*ldlrap1a*) (2.18 FC) are upregulated in the white fillet group muscle.

Within the muscle cell, Astaxanthin is deposited in the myotome and binds with actomyosin to form a complex in salmon [16]. Several genes involved in actomyosin structure organization (GO: 0031032) and members of their families are modulated in the muscle in this study, as stated in the results.

Schmeisser et al. [27] suggested that Astaxanthin recruits coronin for its transport inside muscle cells via actin network polymerization. They found *coronin* to be upregulated in the muscle of Atlantic salmon fed an Astaxanthin-supplemented diet. Similarly, *coronin-2B* (1.61 FC) and *Coronin-1 C* (18.77 FC) are upregulated in this study’s red fillet group muscle.

Two of the most significantly enriched canonical pathways identified in the muscle are the FXR/RXR and LXR/RXR activation pathways. Studies have shown that carotenoids and their metabolites, like retinol and all-trans-retinoic acid (ATRA), can alter gene expression [79, 80] through their interaction with nuclear receptors, including retinoic acid receptor (RAR) and retinoid X receptor (RXR) in human [47, 81, 82]. The LXR/RXR activation pathway is activated in the white versus red fillet group DEGs. This might suggest more availability of retinoids in the white fillet group due to the breakdown of Astaxanthin.

Other top canonical pathways are oxidative phosphorylation and mitochondria dysfunction. The result shows that oxidative phosphorylation is inhibited in the white fillet group, and mitochondrial dysfunction is activated in the same group. The disorder of mitochondrial function in the white fillet group may contribute to the lesser intensity of fillet color observed in the white fillet group. Mitochondrial dysfunction is a precursor to increased oxidative stress caused by the release of ROS (reactive oxygen species) and RNS (reactive nitrogen species). Reactive oxygen species and oxidative stress can deteriorate lipids, adversely affecting fillet color [83, 84]. A GWAS study identified several genes involved in myoglobin homeostasis and protection against lipid oxidation influencing fillet color in rainbow trout [5]. Such genes include *cytochrome b5, ATP synthase subunit β, mitochondrial (ATP5F1B), calsequestrin, peroxiredoxin, superoxide dismutase, sestrin, and ubiquitin carboxyl-terminal hydrolase.* The lysine degradation pathway, a KEGG pathway identified in the muscle, is confined to the mitochondria [85], and mitochondria function can affect fillet color. It is also possible that the lack of carotenoid or reduced concentration of carotenoid in the white fillet group causes mitochondrial dysfunction and the resultant whiteness of the fillet.

Regarding oxidative stress, ascorbate metabolism and FoxO signaling pathways are identified KEGG pathways enriched in the muscle. Ascorbate metabolism can be a free radical scavenger [86]. The FoxO signaling pathway also confers resistance to oxidative stress [87, 88].

Oxidative phosphorylation is identified by both the KEGG pathway and IPA analysis. IPA analysis showed that oxidative phosphorylation is inhibited in the white fillet group. From the identified KEGG and GO terms enriched in the downregulated genes between the white and red fillet group, it is evident that activities in ribosomes and mitochondria are prevalent. Ribosomal activities have been associated with fillet color in fish and skin pigmentation in mice [73–75]. Ribosomal proteins in the proteome were identified as proteins whose change is associated with fillet color in grouper fish and tilapia [74, 75]. The role of mitochondria activity in fillet color change, especially during storage, via their actions on increasing mitochondrial respiration, function oxygen consumption, and metmyoglobin reduction has been reported in various fish species and cattle [74, 89, 90]. Notably, ribosomal and mitochondrial activity pathways are identified in both the liver and muscle DEGs.

The IPA result also shows that there is a disorder of lipid metabolism in the white fillet group. Uptake and accumulation of lipids are inhibited in the white fillet group. As discussed above, carotenoid metabolism and utilization are intricately linked with lipid metabolism; therefore, pathways and functions that inhibit the uptake and accumulation of lipids will likely inhibit the accumulation and use of carotenoids.

Concerning immunity, cellular infiltration by neutrophils, phagocytes, and leukocytes is inhibited in the white fillet group, as identified by the IPA analysis. This suggests an advantage to the red fillet group in having a better immune response. There has been evidence in the literature about the potential benefit of carotenoids in improving the immune system in fish [91–93]. We also observed GO terms of certain biological processes involved in the immune response in the muscle. They include complement activation, humoral immune response, immune effector process, hemostasis, activation of the immune response, blood coagulation, positive regulation of immune response, wound healing, positive regulation of immune system process, and regulation of response to stimulus.

### Important group/class of differentially expressed genes

We identified several DEGs groups that are relevant for carotenoid metabolism. One group is the Apolipoproteins, fatty acid elongases and ABC transporters. Another group is the immune and stress response genes. DEGs within each group and how they aid carotenoid metabolism and modulate stress and immune response are included in supplementary file 4.

In summary, our results show that the ability to absorb and utilize carotenoids confers an advantage in terms of adaptation to stress. Likewise, we observed that several immune genes are modulated in this study, but more studies are needed to investigate whether it confers an advantage to the red fillet group regarding immunity. Some studies on wild salmons with divergent color phenotypes have found no immune-related benefit of carotenoids, while others identified that divergence in color phenotype due to varied carotenoid assimilation resulted in genetic differences in immune-related genes [94].

## Conclusion

We identified significant differences among rainbow trout families in the ability to utilize and retain Astaxanthin. To investigate the genetic differences among families, we applied a transcriptome approach to investigate the molecular mechanisms influencing the absorption and utilization of Astaxanthin in the pyloric caecum, liver, and muscle. We identified several gene pathways involved in lipid/carotenoid metabolism and transport, ribosomal activities, mitochondrial functions, and stress homeostasis. The higher expression of BCO1 and BCO2 in the liver and muscle of the white fillet group agrees with previous studies in other salmonids and our previous GWAS in rainbow trout, indicating that these enzymes metabolize Astaxanthin and reduce its availability for enriching fillet color. In addition to using carotenoid to boost fillet color, several immune-relevant and stress response genes were differentially expressed, suggesting Astaxanthin’s potential to provoke an immune response and be beneficial for managing stress. Also, this study, perhaps for the first time, observed mitochondrial dysfunction, oxidative phosphorylation, and lipid uptake inhibition in the white fillet group, which could contribute to reduced carotenoid absorption and retention-ability. The results from this study deepen our understanding of the carotenoid dynamics in rainbow trout and can lead to strategies to improve Astaxanthin retention in breeding programs, for example, via marker- and genomic selection and manipulation. Achieving this will help meet the consumers’ need for improved fillet quality and increase farmers’ profitability.

### Electronic supplementary material

Below is the link to the electronic supplementary material.

**Additional file 1: Table S1**: Differentially expressed genes (DEGs) in the pyloric caecum, liver, and muscle, and groups of DEGs

**Additional file 2: Table S2**: KEGG and GO terms for DEGs in the liver and muscle

**Additional file 3: Table S3**: Ingenuity Pathway Analysis (IPA) Diseases and Functions

**Supplementary file 4**: Important Group/Class of Differentially Expressed Gene

## Data Availability

All datasets generated for this study are included in the manuscript and/or the Additional Files. All raw sequence data generated in this study has been deposited in NCBI under BioProject accession number: PRJNA974903, https://www.ncbi.nlm.nih.gov/bioproject/PRJNA974903.

## List of abbreviations

DEGs: Differentially Expressed Genes
FC: Fold change
GO: Gene Ontology
IPA: Ingenuity Pathway Analysis
IPKB: Ingenuity pathway knowledge base
KEGG: Kyoto Encyclopedia of Genes and Genomes
NCCCWA: USDA National Center of Cool and Cold Water Aquaculture
PCR: Polymerase Chain Reaction
