# Supplementary material for: RNA-Seq analysis of the pyloric caecum, liver, and muscle reveals molecular mechanisms regulating fillet color in rainbow trout
Source: BMC Genomics. 2023 Sep 28;24:579. doi: 10.1186/s12864-023-09688-5 (PMC10537910; doi:10.1186/s12864-023-09688-5)
Supplement: Supplementary file 4 — Important Group/Class of Differentially Expressed Gene [file 12864_2023_9688_MOESM4_ESM.docx]

**Important Group/Class of Differentially Expressed Genes**

**Apolipoproteins, Fatty acid elongases and ABC transporters**

Carotenoids are transported in the plasma complexed with lipoproteins [1, 2]. Apolipoproteins are a constituent of lipoproteins. They attach to various classes of lipoproteins like the chylomicron, low-density lipoprotein, very low-density lipoprotein, and high-density lipoproteins. They, therefore, aid in the absorption and transport of lipids and carotenoids [3, 4].

*Apolipoprotein B-100* is upregulated in the liver (-6.91, -1.74, -2.38 FC) and pyloric (-9.61 FC) of the red fillet group in this study. Apolipoprotein B-100 is synthesized in the liver and is associated with low-density lipoprotein, very low-density lipoprotein, and intermediate-density lipoprotein [4]. The bulk of the carotenoid transported in the plasma is associated with low-density lipoprotein in humans [1]. This might suggest that the uptake and transport of lipoproteins and carotenoids from the pyloric cecum to the liver and from the liver to the muscle is better in the red fillet group.

In the muscle, the majority of the differentially expressed apolipoproteins identified in this study are downregulated in the red fillet group (supplementary table 1). This pattern of expression might represent the retention of carotenoids in the muscle rather than transport out of the muscle. Only *apolipoprotein O, a* (-2.04 FC) and *apolipoprotein D* (-90.09 FC) are enriched in the muscle of the red fillet group. In summary, it appears that apolipoprotein genes are enriched in the red fillet group within tissues (i.e., pyloric cecum, liver) where transportation through the blood is still needed but downregulated in tissues (i.e., muscle) where export of such carotenoids into the blood is not required.

Another carotenoid transporters implicated in the uptake and/or efflux of carotenoids from various cells are the ABC (ATP binding cassette) family transporters [5, 6]. It has been suggested that the secretion of beta-carotene metabolites into the bloodstream might involve certain basolateral proteins like the ABC transporters that can assist in the efflux of these metabolites [5]. Multidrug resistance-associated protein 9 (2.66 FC), a member of the ABC family, is upregulated in the pyloric of the white fillet group in this study. Upregulation of this gene in the white fillet group might suggest that this gene is not as effective as other ABC transporters.

Several ABC transporter genes are differentially expressed in the liver and muscle in this study. It is of note that most of those genes are upregulated in the red fillet group in the liver (supplementary table 1) : The reverse is the case in the muscle where most of the ABC transporter genes are upregulated in the white fillet group: A plausible explanation for this is that carotenoids in the liver of the red fillet group are better transported through the blood to the muscle, where it gets deposited and retained with no need for further export back to the blood.

*ABCG2* has been identified as an exporter of lipophilic molecules, and Zoric et al. [7] suggested that this protein affects fillet color by exporting astaxanthin from the enterocyte back into the intestinal lumen, thereby reducing astaxanthin availability for deposition in the muscle. They found that *ABCG2-1a* protein was more abundant in the intestine of pale-fleshed Atlantic salmon than in the red-fleshed fish. *ABCG2* can also pump endogenous and exogenous compounds out of cells and tissues [8]. The gene is more enriched in the muscle and liver of the white fillet group in this study, suggesting that the astaxanthin from the white fillet group is transported

**Immune and stress response genes**

**Stress response**

In humans, tumor necrosis factor receptor (TNFR) has been associated with oxidative stress. TNFR1 can directly induce oxidative stress by activating reactive oxygen and nitrogen species (ROS and RNS)-producing enzymes [9]. Mitochondrial dysfunction is also a precursor to increased oxidative stress caused by the release of ROS and RNS. *Tumor necrosis factor receptor superfamily member 14* (4.26 FC), *tumor necrosis factor receptor-associated factor 2* (2.97 FC), *tumor necrosis factor alpha-induced protein 2* (2.46 FC) and *tumor necrosis factor ligand superfamily member 10* (2.25 FC) are all upregulated in the muscle of the white fillet group in this study. Reactive oxygen species and oxidative stress can deteriorate lipids, adversely affecting fillet color [10, 11]. *Tumor necrosis factor* (-9.8 FC) is found to be upregulated in the red fillet group. The same pattern of *TNF* expression was observed in the liver with several TNF genes upregulated in the white filet group and a few TNF genes upregulated in the red fillet group (supplementary table 1). Antioxidants are therefore needed to limit the effect of reactive oxygen species.

Antioxidant-relevant genes like the *glutathione peroxidase* and *glutathione S-transfer*ase are upregulated in the muscle of the red fillet group (supplementary table 1). Peroxidation by ROS is detrimental to bright reddish fillet color [11]. Glutathione peroxidase 1 (*gpx1*) is an antioxidant that clears reactive oxygen species such as superoxide and hydrogen peroxide from the cells, preventing them from causing oxidative damage to DNA, proteins, and membrane lipids [12]. Shabanzadeh et al. [13] also found antioxidant-relevant genes like the *superoxide dismutase, catalase, glutathione peroxidase, glutathione S-transferase and nuclear factor erythroid-2 related factor 2* to be upregulated in the kidney and liver of rainbow trout fed astaxanthin supplemented diet when compared to those without astaxanthin in their diet. They concluded that astaxanthin could ameliorate oxidative stress. Therefore, the red fillet group might have an advantage in their ability to ameliorate oxidative stress.

The upregulated genes in the red fillet group with the highest fold change difference in the muscle are related to stress homeostasis. *Hsp30* (-5.62, -11.59, -53.05, -55.93, -92.8, -135.53, -136.63, -151.27, -92.8, -297.82 FC) and *hsp70*(-3.02 FC). A study on rainbow trout indicated that *hsp30* is expressed in response to heat stress, and its expression can protect cells and tissues against such stressful conditions [14]. Intracellular proteins are usually denigrated into abnormal proteins during stress conditions. Small heat shock proteins, including *hsp30* and *hsp70* expression, can help maintain refolding of the abnormal proteins by binding to such proteins [15]. Heat shock protein 60kD protein 1 (chaperonin, hspd1) was shown to interact with mitochondrial enzymes citrate synthase and malate dehydrogenase to affect tilapia fillet color and beef color [16, 17]. They showed that HSPs expression was negatively corrected with fillet lightness. The nucleotide triphosphate diphosphatase *NUDT15* (-268.46 FC) is one of the most differentially modulated genes upregulated in the muscle of the red fillet group. It functions to protect cells against oxidative stress [18].

Response to cellular stress can be through regular antioxidants like superoxide dismutase and glutathione peroxidase, mopping up free radicals [19]. Heme oxygenase is a member of the heat shock protein family that is also induced during cellular stress [20]. Several researchers have proposed that upregulation of the heme oxygenase is an adaptive mechanism that helps to protect cells from oxidative damage and return cells to a homeostatic state in mammals [21-23]. Heme oxygenase catalyzes the degradation of heme to biliverdin, ferrous ion, and carbon monoxide[20]. Poss & Tonegawa [20] observed that murine cells lacking the heme oxygenase 1 (*Hmox1*) gene are susceptible to accumulation of free radicals and oxidative stress. They concluded that the upregulation of *Hmox1* serves as an adaptive mechanism to protect cells from oxidative damage during stress conditions. Heme oxygenase genes are enriched in the liver: *Heme oxygenase 2-like* (-1.67 FC) and muscle: *heme oxygenase* (-2.5, -2.96 FC), *heme oxygenase 1a* (-3.71 FC) of the red fillet group in this study. This result seems to reflect another beneficial role of astaxanthin in its ability to provide antioxidant capacity. *Heme oxygenase-1* was upregulated in the liver of Atlantic salmon fish fed an astaxanthin-supplemented diet compared to those on a control diet [24]. The author suggested that the fish fed astaxanthin were better able to respond to oxidative stress.

The presence of free heme (non-protein bound heme) can cause lipid peroxidation [25], oxidation of low-density lipoprotein [26, 27], and result in cell death [27]. The cell thus needs protection against heme-catalyzed injuries. Heme-binding proteins functions in this regard to protect molecules and cells against the deleterious effect of free heme [28]. Heme-binding proteins are upregulated in the pyloric: *heme-binding protein 2* (-8.85 FC), liver: *heme-binding protein 1* (-4.45, -1.42 FC), and muscle: *heme-binding protein 1* (-7.71 FC) of the red fillet group in this study.

**Immune response**

Iron metabolism is closely linked with innate immunity because it regulates iron availability. Pathogens require iron for their proliferation and production of virulence factors, and iron metabolism can limit their proliferation by preventing them from having access to iron uptake through iron-withholding strategies [29, 30]. This iron-withholding strategy is controlled by the upregulation of plasma proteins like ferritin that can segregate and convert excess iron into a non-toxic, organic form for storage [31]. This stored iron will be unavailable to pathogens. Several studies have reported that the ferritin-middle sub-unit is activated in response to infection and oxidative stress in fish [32-38]. *Ferritin-middle sub-unit* is upregulated in the hindgut of the Atlantic salmon group with more reddish flesh coloration than the less reddish salmon group [6]. In this study, we observed upregulation of *ferritin, middle subunit* in the pyloric (-8.89 FC), and muscle (-1.96, -2.01, -2.4, -2.85, -3.28, -3.5, -6.53 FC) of the red fillet group. However, ferritin H-3 (147.7 FC) was highly upregulated in the liver of the white fillet group.

Several cathepsin genes (*cathepsin Bb* (-1.68 FC), *cathepsin K* (-1.78, -5.88 FC), *cathepsin B* (-1.92 FC), and *cathepsin L1* (-5.54 FC)) are upregulated in the muscle of the red fillet group in this study. Cathepsin B, K, and L belong to a class of cysteine proteases that confers self-defense against pathogens in fish and assist in immune response in vertebrates [39, 40]. They can also activate proteolysis and muscle degradation in fish [41]. The overrepresentation of the cathepsin genes in the red fillet group may suggest that the red fillet group can better activate its defense mechanism against pathogens. Vo et al.(2021) corroborated [6]this line of thought, where they found upregulation of cathepsin B and L in the hindgut of the Atlantic salmon group with a more reddish flesh color as compared to the less reddish class salmon.

Studies have shown the beneficial effect of carotenoids on immune response. Schmeisser [42] reported that astaxanthin supplementation effects a lowered expression of guanylate-binding protein in the pyloric, which can result to decrease inflammation through lowered interferon (IFN)-gamma production in Atlantic salmon. Studies on rainbow trout reported that carotenoid supplementation reduced mortality and increased protection of the fish when challenged with infectious hematopoietic necrosis virus [43, 44]. They hypothesized that this protection against viral pathogens is achieved via inhibiting virally induced and reactive oxygen species-mediated oxidative stress. Ytrestøyl et al. [24] observed decreased expression of immune-related genes in the intestine of Atlantic salmon deprived of astaxanthin compared to those fed an astaxanthin-supplemented diet but upregulation of immune-related genes in the skeletal muscle. Some of such modulated genes in the intestine include *very large inducible GTPase (vlig), TRIM21-like, TRIM39-like, ubiquitin-protein ligase, ladderlectin, GTPase IMAP family member 4 (gimap4), GTPase IMAP family member 7 (gimap7), and GTPase IMAP family member 8 (gimap8)*. In this study, several GIMAPs genes are modulated in the pyloric, muscle, and liver (supplementary table 1). It is difficult to find a consistent pattern of expression as some GTPase is upregulated in the white fillet group, while others are upregulated in the red fillet group. This might be due to the addition of astaxanthin in the diet of both groups. The GIMAP genes are essential for the development and survival of the mammalian B and T cells and lymphocytes in general [45-48].

Ladderlectin is another differentially expressed immune gene identified in the pyloric: (-4.47 FC) and muscle (-8.15 FC). Ladderlectin has been described as having the ability to get rid of bacteria by binding to bacteria lipopolysaccharides in rainbow trout [49]. They have bacterial and viral pathogen recognition receptors capable of binding to their surfaces and leading to phagocytosis [50, 51]. This gene is upregulated in the pyloric and muscle of the red fillet group in this study. It was also identified as down-regulated in the intestine of Atlantic salmon deprived of astaxanthin compared to those fed an astaxanthin-supplemented diet [24].

Other immune-relevant genes modulated in this study include the TRIM (tripartite mortif-containing protein), CD22, NLR family CARD domain-containing protein, Pancreatic secretory granule membrane major glycoprotein GP2, *Ig mu chain C region membrane-bound form,* NACHT, LRR, and PYD domains-containing protein genes.

Several GO biological process terms involved in immune response are also upregulated in the muscle of the white fillet group in this study.

The most upregulated genes between the white versus red fillet group in the muscle encode digestive enzymes with immune functions: acidic mammalian chitinase (728.07, 575.56 FC) and gastric chitinase (565.2 FC). Chitinases have recently been reported to be involved in innate and adaptive immunity in humans [52-54]. They protect against chitin-containing pathogens by degrading the chitins in their cell wall.

In summary, from our results, it appears that ability to absorb and utilize carotenoids confer an advantage in terms of adaptation to stress. Likewise, we observe that several immune genes are modulated in this study, but more studies are needed to investigate whether it confers an advantage to the red fillet group in terms of immunity or not.

**Parameters used for the Differential Gene Expression Analyses**

**Adapter and low-quality reads trimming parameters**

Quality trim = Yes, Quality limit = 0.05, Trim adapter list = Illumina_New trim adapter list, Automatic read-through adapter trimming = Yes, Trim homopolymers from 5' = Yes, Trim homopolymers from 3' = Yes, polyA = Yes, polyT = Yes, Remove 5' terminal nucleotides = No, Remove 3' terminal nucleotides = No, Fixed length trimming = No, Maximum length = 150, Trim from side = 3'-end, Discard short reads = No, Discard long reads = No, Save discarded sequences = Yes, Save broken pairs = No, Create report = Yes

**Reads Mapping parameters.**

Reference type = Genome annotated with genes and transcripts, Reference sequence = GCF_013265735.2_USDA_OmykA_1.1_genomic (Genome), Gene track GCF_013265735.2_USDA_OmykA_1.1_genomic (Gene), mRNA track = GCF_013265735.2_USDA_OmykA_1.1_genomic (mRNA), Use spike-in controls = no, Mismatch cost = 2, Insertion cost = 3, Deletion cost = 3, Length fraction = 0.8, Similarity fraction = 0.8, Global alignment = Yes, Strand specific = Both, Library type = Bulk, Maximum number of hits for a read = 20, Count paired reads as two = No, Ignore broken pairs = No, Expression value = RPKM, Calculate expression for genes without transcripts = Yes, Create reads track = No, Create report = Yes, Create fusion gene table = No, Create list of unmapped reads = No

**Differential Expression Analysis Parameters**

Technology = Whole transcriptome RNA-Seq, Filter on average expression for FDR correction = No Metadata table = Metadata_Table_Colo_Liver, Test differential expression due to = Genetic_Line, While controlling for = Not set, Comparisons = All group pairs, Metadata table encoded as a tsv = Not set

1. Lowe GM, Bilton RF, Davies IG, Ford TC, Billington D, Young AJ: **Carotenoid composition and antioxidant potential in subfractions of human low-density lipoprotein**. *Annals of clinical biochemistry* 1999, **36**(3):323-332.

2. Shete V, Costabile BK, Kim Y-K, Quadro L: **Low-density lipoprotein receptor contributes to β-carotene uptake in the maternal liver**. *Nutrients* 2016, **8**(12):765.

3. Ribalta J, Vallvé J-C, Girona J, Masana L: **Apolipoprotein and apolipoprotein receptor genes, blood lipids and disease**. *Current Opinion in Clinical Nutrition & Metabolic Care* 2003, **6**(2):177-187.

4. Feingold KR: **Introduction to lipids and lipoproteins**. *endotext [internet]* 2021.

5. Harrison EH: **Mechanisms involved in the intestinal absorption of dietary vitamin A and provitamin A carotenoids**. *Biochimica et Biophysica Acta (BBA)-Molecular and Cell Biology of Lipids* 2012, **1821**(1):70-77.

6. Vo TTM, Nguyen TV, Amoroso G, Ventura T, Elizur A: **Deploying new generation sequencing for the study of flesh color depletion in Atlantic Salmon (Salmo salar)**. *BMC genomics* 2021, **22**(1):1-21.

7. Zoric N: **Characterization of genes and gene products influencing carotenoid metabolism in Atlantic salmon**. 2017.

8. Yuan G, Ma B, Yuan W, Zhang Z, Chen P, Ding X, Feng L, Shen X, Chen S, Li G: **Histone H2A ubiquitination inhibits the enzymatic activity of H3 lysine 36 methyltransferases**. *Journal of Biological Chemistry* 2013, **288**(43):30832-30842.

9. Fischer R, Maier O: **Interrelation of oxidative stress and inflammation in neurodegenerative disease: role of TNF**. *Oxidative medicine and cellular longevity* 2015, **2015**.

10. Scaife J, Onibi G, Murray I, Fletcher T, Houlihan D: **Influence of a-tocopherol acetate on the short-and long-term storage properties of fillets from Atlantic salmon Salmo salar fed a high lipid diet**. *Aquaculture Nutrition* 2000, **6**(1):65.

11. Sampels S: **Oxidation and antioxidants in fish and meat from farm to fork**. *Food industry* 2013:114-144.

12. Lubos E, Loscalzo J, Handy DE: **Glutathione peroxidase-1 in health and disease: from molecular mechanisms to therapeutic opportunities**. 2011.

13. Shabanzadeh S, Vatandoust S, Hosseinifard SM, Sheikhzadeh N, Shahbazfar A-A: **Dietary astaxanthin (Lucantin® Pink) mitigated oxidative stress induced by diazinon in rainbow trout (Oncorhynchus mykiss)**. In: *Veterinary Research Forum: 2023*: Faculty of Veterinary Medicine, Urmia University; 2023.

14. Liu X, Shi H, Liu Z, Wang J, Huang J: **Effect of heat stress on heat shock protein 30 (Hsp30) mRNA expression in rainbow trout (Oncorhynchus mykiss)**. *Turkish Journal of Fisheries and Aquatic Sciences* 2019, **19**(8):681-688.

15. Nakamoto H, Vigh L: **The small heat shock proteins and their clients**. *Cellular and Molecular Life Sciences* 2007, **64**(3):294-306.

16. Xiang H, Sun S, Huang H, Hao S, Li L, Yang X, Chen S, Wei Y, Cen J, Pan C: **Proteomics study of mitochondrial proteins in tilapia red meat and their effect on color change during storage**. *Food Chemistry* 2023, **400**:134061.

17. Kim NK, Cho S, Lee SH, Park HR, Lee CS, Cho YM, Choy YH, Yoon D, Im SK, Park EW: **Proteins in longissimus muscle of Korean native cattle and their relationship to meat quality**. *Meat Science* 2008, **80**(4):1068-1073.

18. Valerie NC: **Roles of NUDT5 and NUDT15 beyond oxidized nucleotide sanitation and their potential as therapeutic targets**: Karolinska Institutet (Sweden); 2018.

19. Coyle JT, Puttfarcken P: **Oxidative stress, glutamate, and neurodegenerative disorders**. *Science* 1993, **262**(5134):689-695.

20. Poss KD, Tonegawa S: **Reduced stress defense in heme oxygenase 1-deficient cells**. *Proceedings of the National Academy of Sciences* 1997, **94**(20):10925-10930.

21. Balla J, Jacob HS, Balla G, Nath K, Eaton JW, Vercellotti GM: **Endothelial-cell heme uptake from heme proteins: induction of sensitization and desensitization to oxidant damage**. *Proceedings of the National Academy of Sciences* 1993, **90**(20):9285-9289.

22. Otterbein L, Sylvester SL, Choi A: **Hemoglobin provides protection against lethal endotoxemia in rats: the role of heme oxygenase-1**. *American journal of respiratory cell and molecular biology* 1995, **13**(5):595-601.

23. Nath KA, Balla G, Vercellotti GM, Balla J, Jacob HS, Levitt M, Rosenberg ME: **Induction of heme oxygenase is a rapid, protective response in rhabdomyolysis in the rat**. *The Journal of clinical investigation* 1992, **90**(1):267-270.

24. Ytrestøyl T, Afanasyev S, Ruyter B, Hatlen B, Østbye T-K, Krasnov A: **Transcriptome and functional responses to absence of astaxanthin in Atlantic salmon fed low marine diets**. *Comparative Biochemistry and Physiology Part D: Genomics and Proteomics* 2021, **39**:100841.

25. Gutteridge J, Smith A: **Antioxidant protection by haemopexin of haem-stimulated lipid peroxidation**. *Biochemical Journal* 1988, **256**(3):861-865.

26. Li W, Östblom M, Xu LH, Hellsten A, Leanderson P, Liedberg B, Brunk UT, Eaton JW, Yuan XM: **Cytocidal effects of atheromatous plaque components: the death zone revisited**. *The FASEB Journal* 2006, **20**(13):2281-2290.

27. Balla G, Vercellotti G, Muller-Eberhard U, Eaton J, Jacob H: **Exposure of endothelial cells to free heme potentiates damage mediated by granulocytes and toxic oxygen species**. *Laboratory investigation; a journal of technical methods and pathology* 1991, **64**(5):648-655.

28. Gáll T, Pethő D, Nagy A, Hendrik Z, Méhes G, Potor L, Gram M, Åkerström B, Smith A, Nagy P: **Heme induces endoplasmic reticulum stress (HIER stress) in human aortic smooth muscle cells**. *Frontiers in physiology* 2018, **9**:1595.

29. Posey JE, Gherardini FC: **Lack of a role for iron in the Lyme disease pathogen**. *Science* 2000, **288**(5471):1651-1653.

30. Ong ST, Ho JZS, Ho B, Ding JL: **Iron-withholding strategy in innate immunity**. *Immunobiology* 2006, **211**(4):295-314.

31. Arosio P, Levi S: **Cytosolic and mitochondrial ferritins in the regulation of cellular iron homeostasis and oxidative damage**. *Biochimica et Biophysica Acta (BBA)-General Subjects* 2010, **1800**(8):783-792.

32. Lee J-H, Pooley NJ, Mohd-Adnan A, Martin SA: **Cloning and characterisation of multiple ferritin isoforms in the Atlantic salmon (Salmo salar)**. *PLoS One* 2014, **9**(7):e103729.

33. LeBlanc F, Laflamme M, Gagne N: **Genetic markers of the immune response of Atlantic salmon (Salmo salar) to infectious salmon anemia virus (ISAV)**. *Fish & Shellfish Immunology* 2010, **29**(2):217-232.

34. Robertson LS, McCormick SD: **The effect of nonylphenol on gene expression in Atlantic salmon smolts**. *Aquatic toxicology* 2012, **122**:36-43.

35. Peatman E, Baoprasertkul P, Terhune J, Xu P, Nandi S, Kucuktas H, Li P, Wang S, Somridhivej B, Dunham R: **Expression analysis of the acute phase response in channel catfish (Ictalurus punctatus) after infection with a Gram-negative bacterium**. *Developmental & Comparative Immunology* 2007, **31**(11):1183-1196.

36. Neves JV, Wilson JM, Rodrigues PN: **Transferrin and ferritin response to bacterial infection: the role of the liver and brain in fish**. *Developmental & Comparative Immunology* 2009, **33**(7):848-857.

37. Wang W, Zhang M, Sun L: **Ferritin M of Cynoglossus semilaevis: an iron-binding protein and a broad-spectrum antimicrobial that depends on the integrity of the ferroxidase center and nucleation center for biological activity**. *Fish & Shellfish Immunology* 2011, **31**(2):269-274.

38. Zheng W-j, Hu Y-h, Sun L: **Identification and analysis of a Scophthalmus maximus ferritin that is regulated at transcription level by oxidative stress and bacterial infection**. *Comparative Biochemistry and Physiology Part B: Biochemistry and Molecular Biology* 2010, **156**(3):222-228.

39. Subramanian S, MacKinnon SL, Ross NW: **A comparative study on innate immune parameters in the epidermal mucus of various fish species**. *Comparative Biochemistry and Physiology Part B: Biochemistry and Molecular Biology* 2007, **148**(3):256-263.

40. Zhou Z-j, Qiu R, Zhang J: **Molecular characterization of the cathepsin B of turbot (Scophthalmus maximus)**. *Fish Physiology and Biochemistry* 2015, **41**:473-483.

41. Gaarder M, Bahuaud D, Veiseth-Kent E, Mørkøre T, Thomassen M: **Relevance of calpain and calpastatin activity for texture in super-chilled and ice-stored Atlantic salmon (Salmo salar L.) fillets**. *Food Chemistry* 2012, **132**(1):9-17.

42. Schmeisser J, Verlhac-Trichet V, Madaro A, Lall SP, Torrissen O, Olsen RE: **Molecular Mechanism Involved in Carotenoid Metabolism in Post-Smolt Atlantic Salmon: Astaxanthin Metabolism During Flesh Pigmentation and Its Antioxidant Properties**. *Marine Biotechnology* 2021, **23**(4):653-670.

43. Chang MX, Xiong F: **Astaxanthin and its effects in inflammatory responses and inflammation-associated diseases: recent advances and future directions**. *Molecules* 2020, **25**(22):5342.

44. Amar EC, Kiron V, Akutsu T, Satoh S, Watanabe T: **Resistance of rainbow trout Oncorhynchus mykiss to infectious hematopoietic necrosis virus (IHNV) experimental infection following ingestion of natural and synthetic carotenoids**. *Aquaculture* 2012, **330**:148-155.

45. Saunders A, Webb LM, Janas ML, Hutchings A, Pascall J, Carter C, Pugh N, Morgan G, Turner M, Butcher GW: **Putative GTPase GIMAP1 is critical for the development of mature B and T lymphocytes**. *Blood, The Journal of the American Society of Hematology* 2010, **115**(16):3249-3257.

46. Webb L, Datta P, Bell SE, Kitamura D, Turner M, Butcher GW: **GIMAP1 is essential for the survival of naive and activated B cells in vivo**. *The Journal of Immunology* 2016, **196**(1):207-216.

47. Datta P, Webb LM, Avdo I, Pascall J, Butcher GW: **Survival of mature T cells in the periphery is intrinsically dependent on GIMAP1 in mice**. *European journal of immunology* 2017, **47**(1):84-93.

48. Schwefel D, Arasu BS, Marino SF, Lamprecht B, Köchert K, Rosenbaum E, Eichhorst J, Wiesner B, Behlke J, Rocks O: **Structural insights into the mechanism of GTPase activation in the GIMAP family**. *Structure* 2013, **21**(4):550-559.

49. Hoover GJ, El-Mowafi A, Simko E, Kocal TE, Ferguson HW, Hayes MA: **Plasma proteins of rainbow trout (Oncorhynchus mykiss) isolated by binding to lipopolysaccharide from Aeromonas salmonicida**. *Comparative Biochemistry and physiology part B: biochemistry and molecular biology* 1998, **120**(3):559-569.

50. Young KM, Russell S, Smith M, Huber P, Ostland VE, Brooks AS, Hayes MA, Lumsden JS: **Bacterial-binding activity and plasma concentration of ladderlectin in rainbow trout (Oncorhynchus mykiss)**. *Fish & Shellfish Immunology* 2007, **23**(2):305-315.

51. Reid A, Young K, Lumsden J: **Rainbow trout Oncorhynchus mykiss ladderlectin, but not intelectin, binds viral hemorrhagic septicemia virus IVb**. *Diseases of aquatic organisms* 2011, **95**(2):137-143.

52. Di Rosa M, Brundo VM, Malaguarnera L: **New insights on chitinases immunologic activities**. *World Journal of Immunology* 2016, **6**(2):96-104.

53. Kumar A, Zhang KY: **Human chitinases: structure, function, and inhibitor discovery**. *Targeting Chitin-containing Organisms* 2019:221-251.

54. Hu C, Ma Z, Zhu J, Fan Y, Tuo B, Li T, Liu X: **Physiological and pathophysiological roles of acidic mammalian chitinase (CHIA) in multiple organs**. *Biomedicine & Pharmacotherapy* 2021, **138**:111465.
